# Supplementary material for: Dual-resolving of positional and geometric isomers of C=C bonds via bifunctional photocycloaddition-photoisomerization reaction system
Source: Nat Commun. 2022 May 12;13:2652. doi: 10.1038/s41467-022-30249-z (PMC9098869; doi:10.1038/s41467-022-30249-z)
Supplement: Supplementary file 1 — Supplementary Information [file 41467_2022_30249_MOESM1_ESM.pdf]

Supplementary Information

Dual-Resolving of Positional and Geometric Isomers of C=C Bonds *via* Bifunctional Photocycloaddition-Photoisomerization Reaction System

Guifang Feng,[1] Ming Gao,[1] Liwei Wang,[1] Jiayi Chen,[2] Menglu Hou,[1] Qiongqiong Wan,[1] Yun Lin,[2] Guoyong Xu,[1] Xiaotian Qi,[3] and Suming Chen*[1]

[1]The Institute for Advanced Studies, Wuhan University, Wuhan, Hubei 430072, China.

[2]Department of Anesthesiology, Union Hospital, Tongji Medical College, Huazhong University of Science and Technology, Wuhan, Hubei 430022, China.

[3]Engineering Research Center of Organosilicon Compounds & Materials, Ministry of Education, College of Chemistry and Molecular Sciences, Wuhan University, Wuhan, Hubei 430072, China.

*Email: sm.chen@whu.edu.cn

Supplementary Methods

Gas chromatography mass spectrometric analysis. Gas chromatography mass spectrometric (GC-MS) analysis was carried out by a gas chromatograph (Agilent GC-2030) coupled to a mass spectrometer (Agilent GCMS-QP2020 NX), using a Rtx-5MS fused silica column (30 m × 0.25 mm, 0.25 μm film thickness). The experiments were performed according to the following conditions: injector temperature, 280 °C; injection in spitless mode; gas flow, 0.6 mL/min; and injection volume, 8 μL. The oven temperature was programmed as follows: initial temperature 140 °C, hold for 1 min; ramp at 10 °C/min up to 180 °C, hold for 20 min, then ramp at 0.5 °C/min up to 200 °C, hold for 20 min, finally ramp at 5 °C/min up to 230 °C, hold for 2 min. The total analysis time was 89 min. The single quadrupole mass spectrometer was operated in the full scan mode, with the instrumental temperatures set at 250, 250, and 180 °C for transfer line, source, and quadrupole, respectively. The electron energy was set at 70 eV, data acquisition was carried out in an *m/z* range from 45 to 550 and with a solvent delay for 4.5 min.

The free fatty acids were methylated with 3 mL 0.5 mol/L H_2SO_4-MeOH at 90 °C for 60 min. After the reaction, 3 mL 0.4 mol/L NaOH-MeOH was added for neutralization of the solutions. Then, 5 mL saturated normal saline and 2 mL hexane were added in sequentially for promoting liquid-liquid stratification and extracting the methylated fatty acids. The hexane phase was transferred into a new centrifuge tube for drying with anhydrous sodium sulfate. The obtained solution was concentrated with nitrogen blowing. Finally, the residual was redissolved with 200 uL hexane and centrifuged at 7400 g for 10 min for the analysis of GC-MS.

Data analysis. Thermo Xcalibur (version 4.1) and otofControl (version 6.2) software were used for extracting target ion and calculate its peak area from the mass spectrometric data generated by LTQ-Orbitrap Elite mass spectrometer (Thermo Scientific, Germany) and timsTOF Pro mass spectrometer (Bruker Daltonics, Germany), respectively. MS-DIAL version 4.12 was used for the process of LC-MS data for qualitative analysis. The raw data must be transformed into Abf. files. More details were presented at http://prime.psc.riken.jp/compms/msdial/main.html. The acquired GC-MS data were processed with GCMS solution Version 5.2 (Agilent, USA). The mass spectra matching was processed with a standard Library NIST11.

Computational methods. All density functional theory (DFT) calculations were carried out using the Gaussian 16 software package.[2] The geometries were optimized using the M06-2X functional[3] with a basis set of 6-31G(d) for all atoms. Vibrational frequency calculations were performed for all the stationary points to confirm if each optimized structure is a local minimum or a transition state structure. Truhlar's quasi-harmonic corrections[4] were applied for entropy calculations with 100 cm^{-1} as the frequency cutoff using the Goodvibes program (version 3.0.1).[5] Intrinsic reaction coordinate (IRC) calculations were performed to ensure that the saddle points located were transition states connecting the reactants and the products. Solvation energy corrections were calculated in acetonitrile solvent with the SMD continuum solvation model[6] based on the gas phase optimized geometries. The M06-2X functional with a basis set of 6-311+G(d,p) for all atoms was used for single-point energy calculations.

Materials

Fatty acid standards including FA 14:0, FA 16:1 (9*Z*), FA 16:1 (9*E*), FA 16:0, FA 17:1 (10*Z*), FA 17:1 (10*E*), FA 17:0, FA 18:0, FA 18:1 (9*Z*), FA 18:1 (9*E*), FA 18:1 (11*Z*), FA 18:1 (6*Z*), 18:2 (9*Z*, 11*E*), FA 18:2 (9*Z*, 12*Z*), FA 19:1 (10*Z*), FA 19:1 (10*E*), FA 19:0, FA 20:1 (11*Z*), FA 20:1 (11*E*) and FA 20:4 (5*Z*, 8*Z*, 11*Z*, 14*Z*), were purchased from Sigma-Aldrich. Glycerol phospholipids and glycerides were purchased from Avanti Polar Lipids (Alabaster, AL, USA), including PC 18:1 (9*Z*)/18:1 (9*Z*), PC 18:1 (9*E*)/18:1 (9*E*), PC 18:0/18:2 (9*Z*, 12*Z*), PC 16:0/18:1 (9*Z*), PC 18:1 (9*Z*)/16:0, DG 16:0/18:1 (9*Z*), TG 18:1 (9*Z*)/18:1 (9*Z*)/18:1 (9*Z*), TG 18:2 (9*Z*, 12*Z*)/18:2 (9*Z*, 12*Z*)/18:2 (9*Z*, 12*Z*). The lipid nomenclature introduced by Liebisch et al.[1] is adapted in this study. The notations for double bond positions counted from the carboxyl terminal, where the carbon atom was noted “1”. Consequently, a fatty acid with 18 carbon atoms and one *cis*-C=C between 9th and 10th carbon was abbreviated as FA 18:1 (9*Z*). Methyl benzoylformate and Ir[dFppy]$_2$(dtbbpy)PF$_6$ was purchased from Sigma-Aldrich. Other solvents were purchased from Innochem (Beijing, China) and meet or exceed the analytical grade standard.

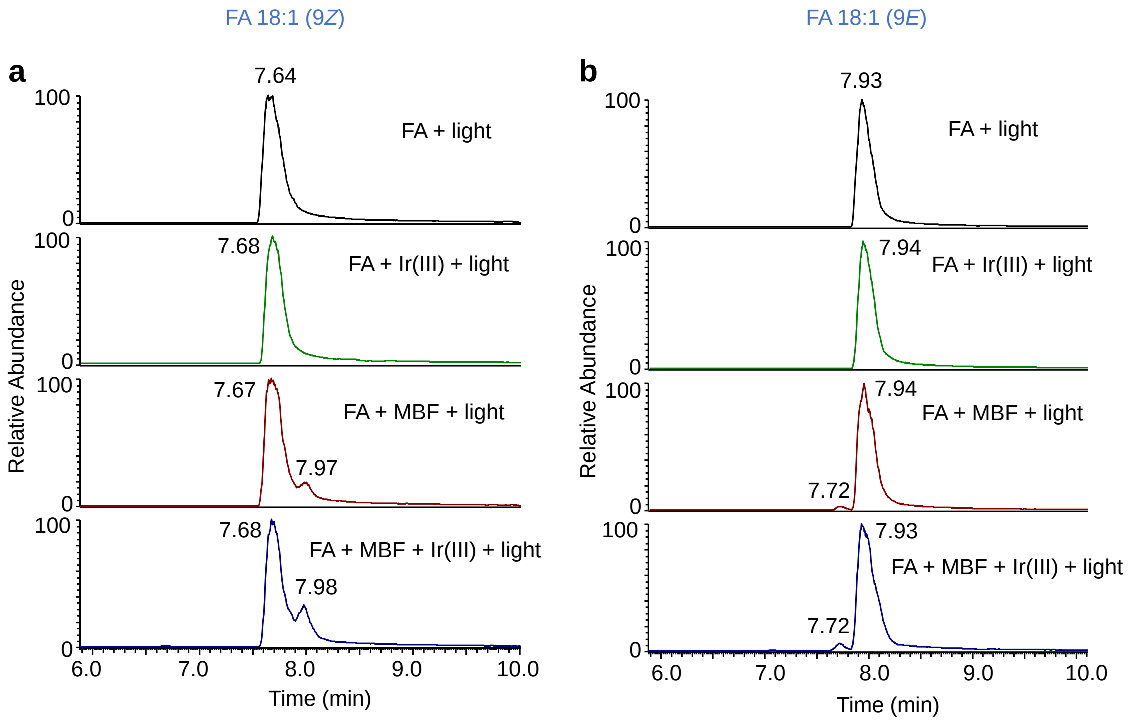

Supplementary Figure 1. EICs of FA 18:1 at different reaction conditions. (a)-(b) EICs of (a) FA 18:1 (9*Z*) and (b) FA 18:1 (9*E*) at reaction conditions of only LED (450 nm) light irradiation, light irradiation in the presence of Ir[dFppy]$_2$(dtbbpy)PF$_6$ (Ir(III)) photocatalyst, light irradiation in the presence of methyl benzoylformate (MBF) and light irradiation in the presence of both MBF and Ir(III) photocatalyst. Reaction time: 10 min.

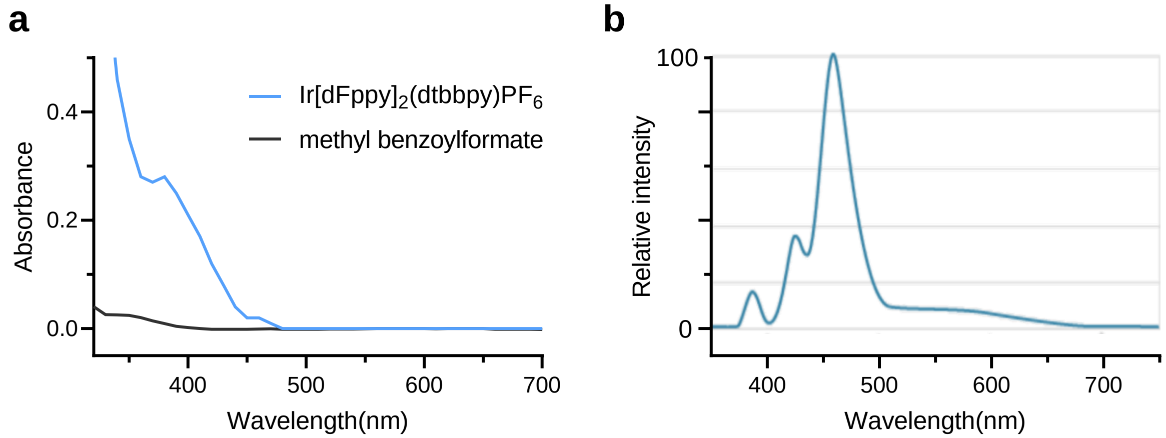

Supplementary Figure 2. Spectroscopic characterization of the reactant, photocatalyst and LED light. (a) Ultraviolet-visible absorption spectra of the solutions of photocatalyst Ir[dFppy]$_2$(dtbbpy)PF$_6$ (0.1 mM) and methyl benzoylformate (1 mM) in MeOH. (b) Full spectrum of the Kessil A160WE-TB blue LED light adopted from vendor’s website.

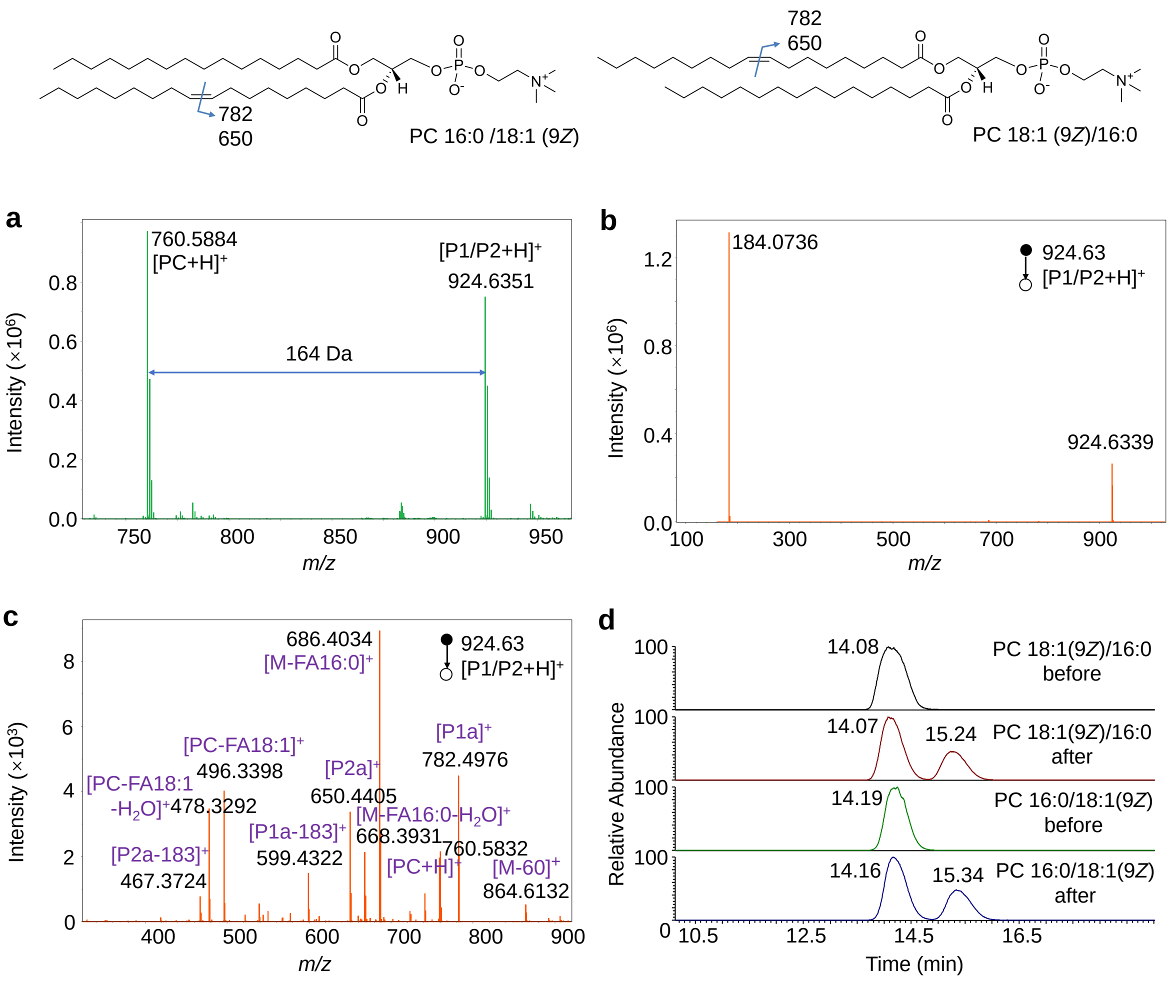

Supplementary Figure 3. Demonstration of the bifunctional reaction system for the identification of C=C bonds location and *E*-*Z* configuration in PC 16:0/18:1 (9*Z*) and 18:1 (9*Z*)/16:0. (a) Mass spectrum of the photocatalytic reaction solution of PC 16:0/18:1 (9*Z*) in positive ion mode. (b)-(c) MS/MS spectrum of the protonated photocycloaddition product P1/P2 in positive ion mode in the mass range of (b) 100-1000 Da and (c) 200-900 Da. (d) Extracted ion chromatograms (EIC) of PC 18:1 (9*Z*)/16:0 and 16:0/18:1 (9*Z*) before and after the photocatalytic reaction in positive ion mode.

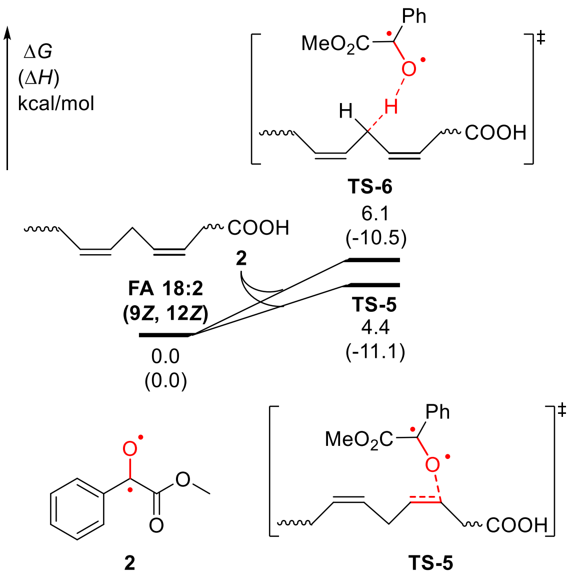

Supplementary Figure 4. Computational study of the comparison between hydrogen atom abstraction and radical addition to alkene in the case of FA18:2 (9*Z*, 12*Z*).

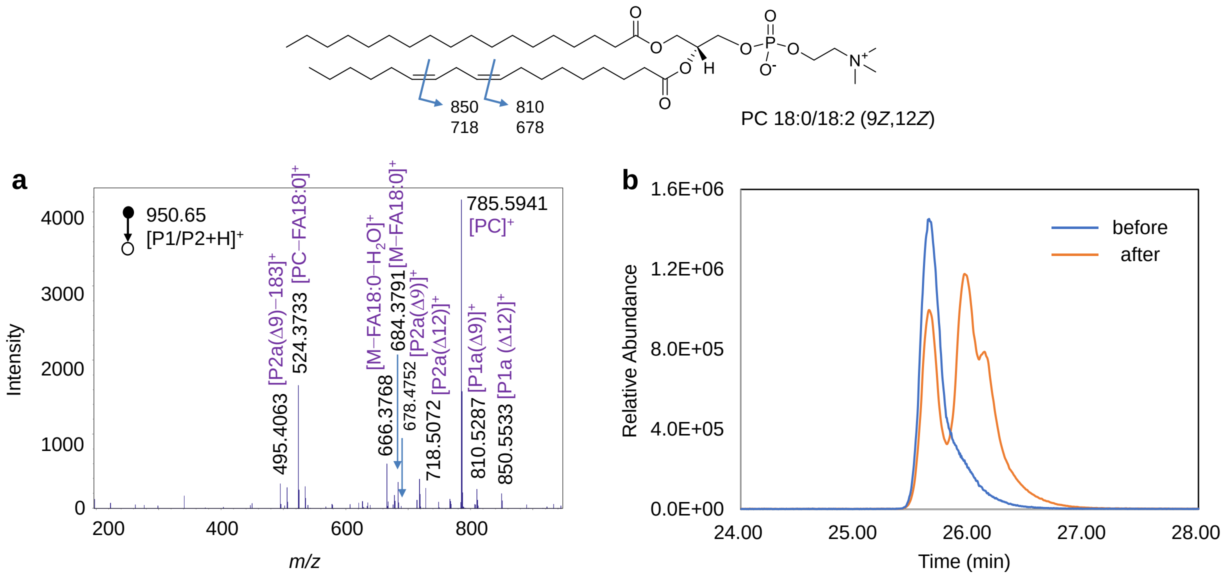

Supplementary Figure 5. Demonstration of the bifunctional reaction system for the identification of C=C bonds location and *E*-*Z* configuration in PC 18:0/18:2 (9*Z*, 12*Z*). (a) MS/MS spectrum of the protonated photocycloaddition product (P1/P2) of PC 18:0/18:2 (9*Z*, 12*Z*). (b) EICs of PC 18:0/18:2 (9*Z*, 12*Z*) before and after the photocatalytic reaction at *m/z* 786.61.

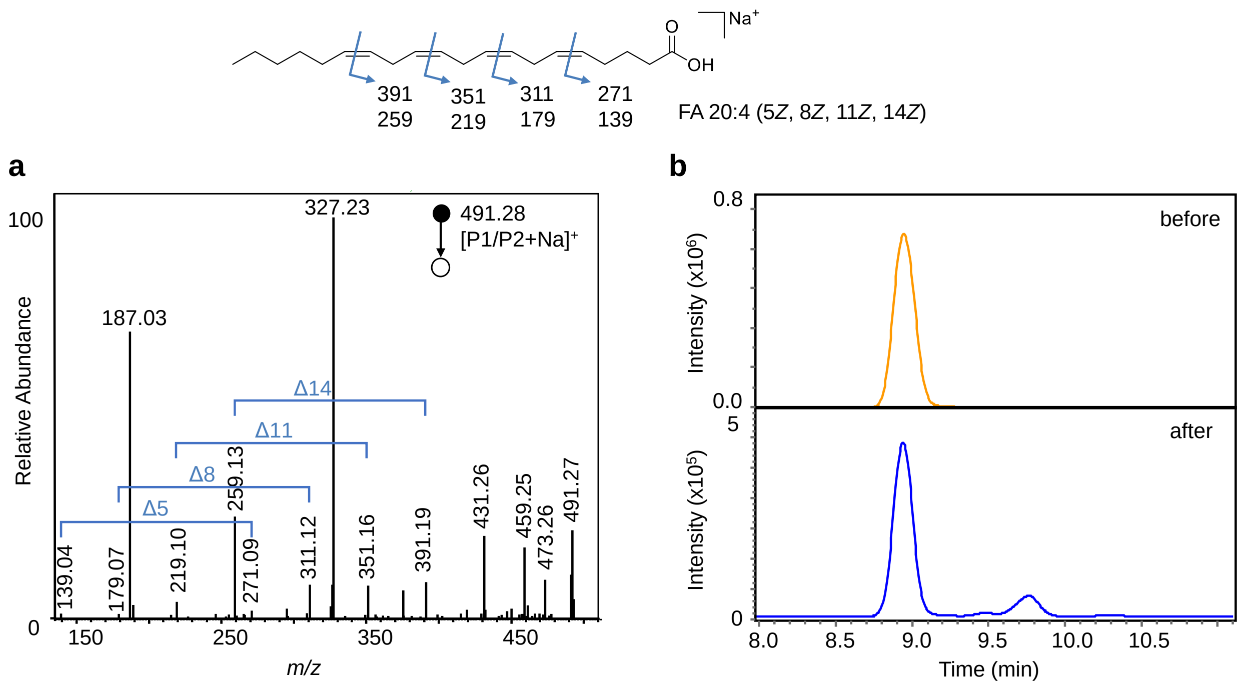

Supplementary Figure 6. Demonstration of the bifunctional reaction system for the identification of C=C bonds location and *E*-*Z* configuration in FA 20:4 (5*Z*, 8*Z*, 11*Z*, 14*Z*). (a) MS/MS spectrum of the sodiated photocycloaddition product (P1/P2) at *m/z* 491.28. (b) EICs of FA 20:4 (5*Z*, 8*Z*, 11*Z*, 14*Z*) before and after the photocatalytic reaction at *m/z* 467.28 in negative ion mode.

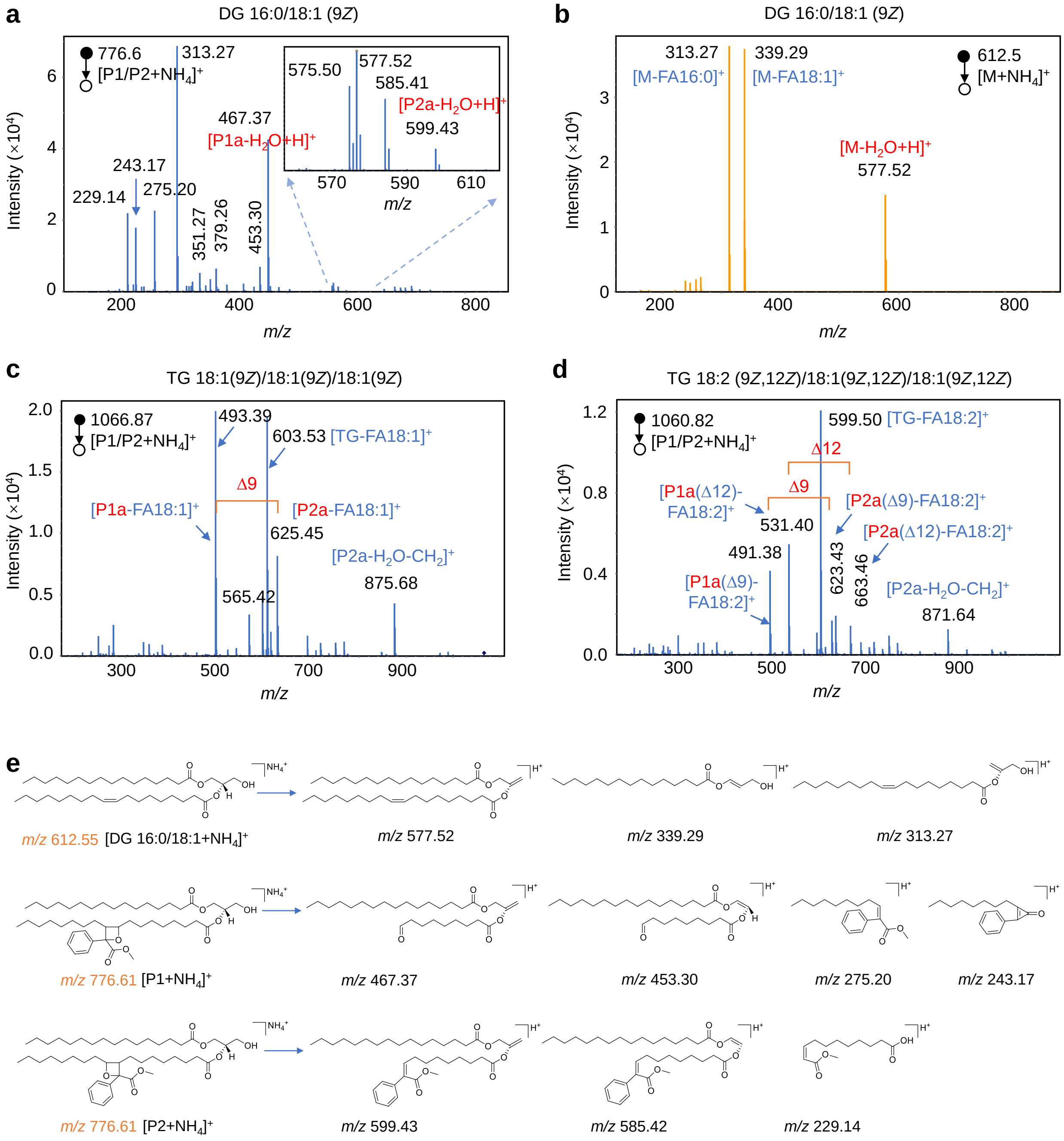

Supplementary Figure 7. Identification of C=C bonds in glycerides. (a)-(d) MS/MS spectrum of the photocycloaddition products of (a) DG 16:0/18:1 (9*Z*), (c) TG 18:1 (9*Z*)/18:1 (9*Z*)/18:1 (9*Z*), (d) TG 18:2 (9*Z*,12*Z*)/18:2 (9*Z*,12*Z*)/18:2 (9*Z*,12*Z*), and (b) MS/MS spectrum of DG 16:0/18:1 (9*Z*) before the reaction. (e) Possible fragmentation pathways for DG 16:0/18:1 (9*Z*) and its cycloaddition products.

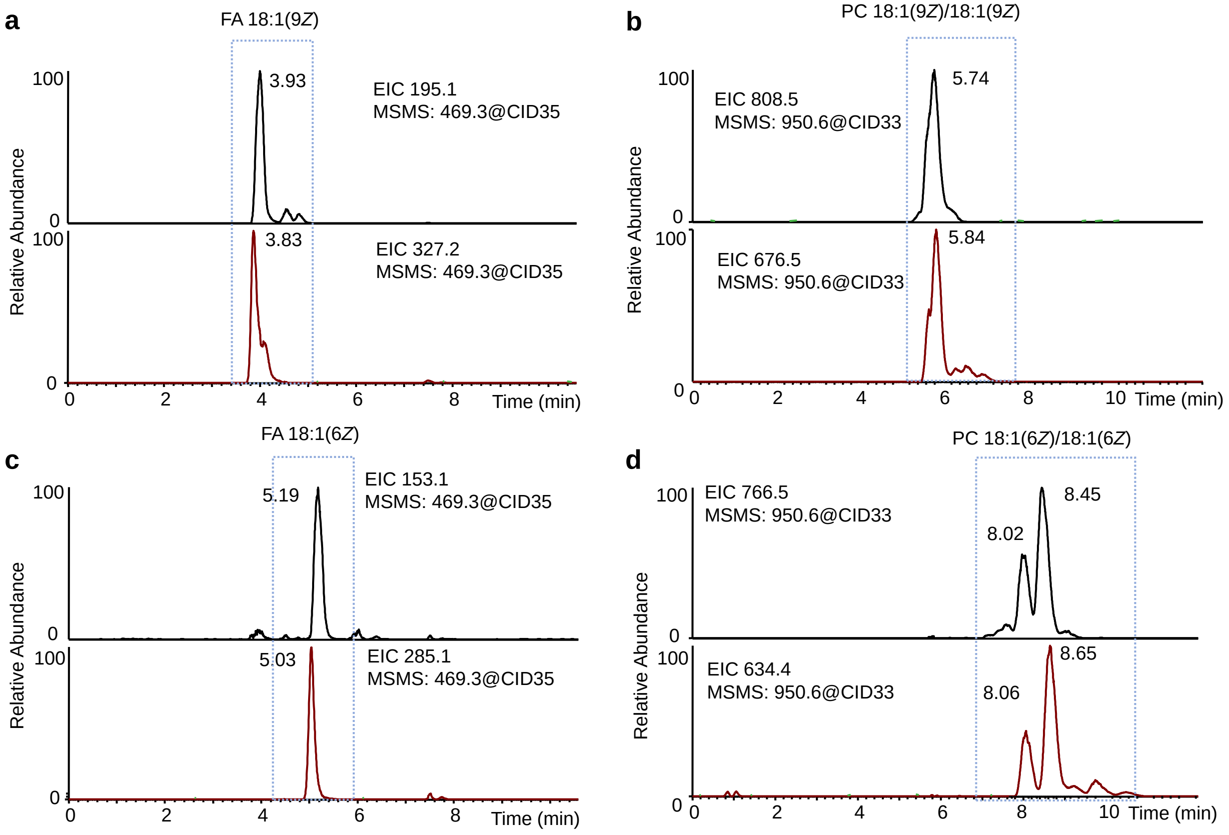

Supplementary Figure 8. EICs of diagnostic ions in tandem MS spectra of photocycloaddition products of (a) FA 18:1 (9*Z*), (b) PC 18:1 (9*Z*)/18:1 (9*Z*), (c) FA 18:1 (6*Z*), and (d) PC 18:1 (6*Z*)/18:1 (6*Z*) in the positive ion mode for quantitative analysis.

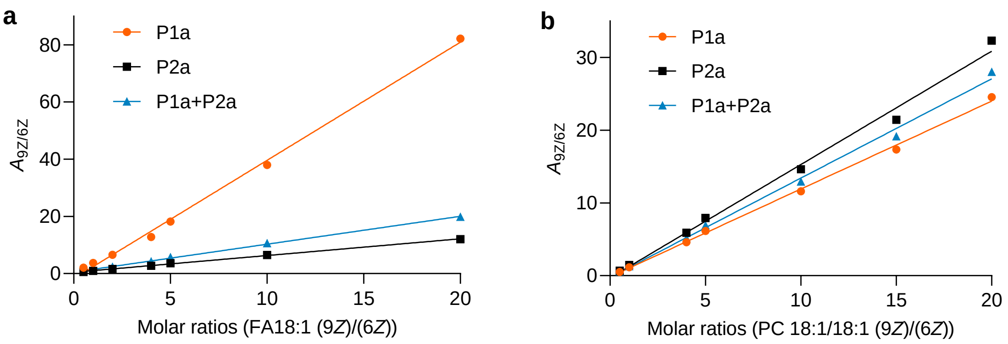

P1a: $y = 0.5855x + 0.4519$, $R^2 = 0.9986$
P2a: $y = 4.1312x - 1.7198$, $R^2 = 0.9973$
P1a+P2a: $y = 0.9755x + 0.5144$, $R^2 = 0.9987$

P1a: $y = 1.5582x - 0.3106$, $R^2 = 0.9931$
P2a: $y = 1.2078x - 0.1689$, $R^2 = 0.9981$
P1a+P2a: $y = 1.3646x - 0.2386$, $R^2 = 0.9959$

Supplementary Figure 9. Quantitative analysis of the C=C positional isomers. (a) Linear relationship established between the peak area ratio (A_{9Z}/A_{6Z}) of the diagnostic ions and molar ratio (C_{9Z}/C_{6Z}) of the two FA 18:1 C=C location isomers. (b) Linear relationship established between the peak area ratio (A_{9Z}/A_{6Z}) of the diagnostic ions and molar ratio (C_{9Z}/C_{6Z}) of the two PC 18:1/18:1 C=C location isomers.

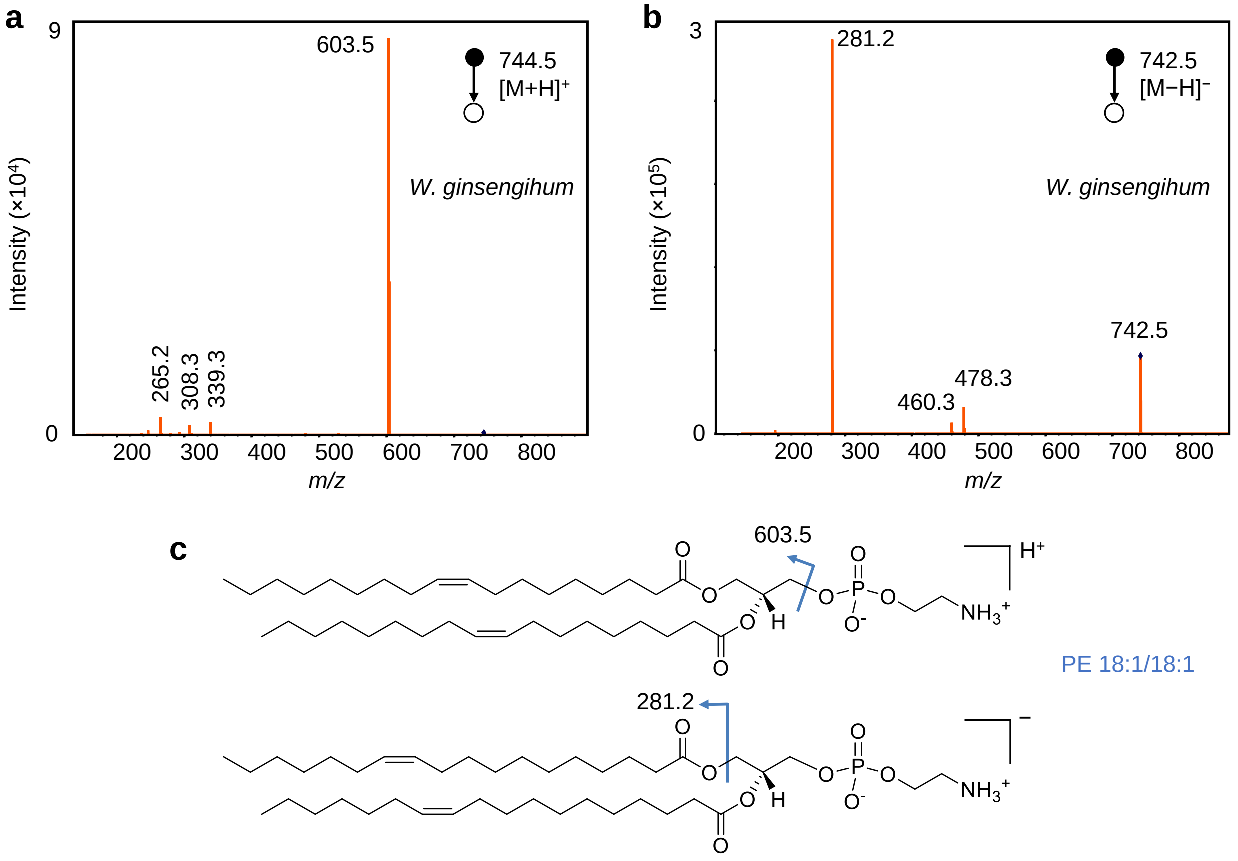

Supplementary Figure 10. Analysis of the structure of PE 36:2 by tandem MS. (a) MS/MS spectrum of $[M+H]^+$ at *m/z* 744.5 in positive ion mode. (b) MS/MS spectrum of $[M-H]^-$ at *m/z* 742.5 in negative ion mode. (c) Structures of PE 18:1 (Δ9)_18:1 (Δ9) ions and possible fragmentation pathways in positive and negative ion modes, respectively.

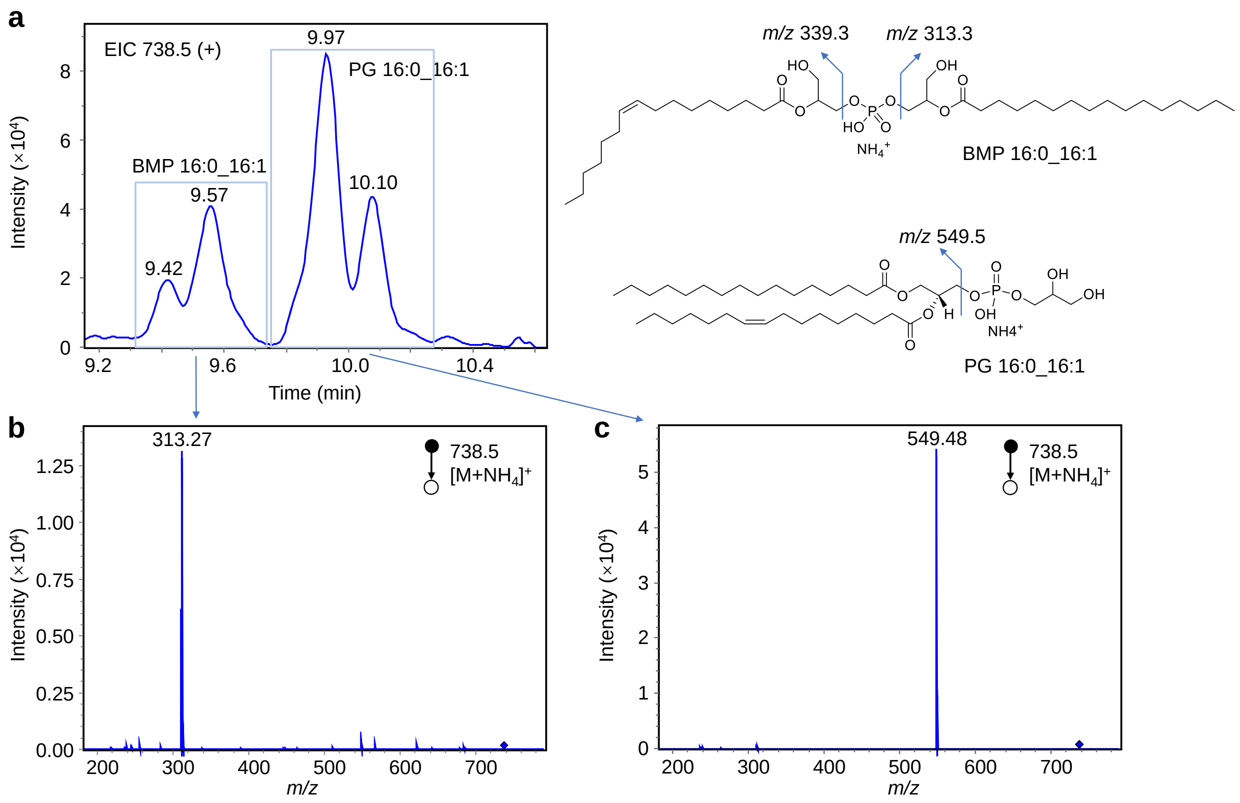

Supplementary Figure 11. Identification of bis(monoacylglycero)phosphate (BMP) in *P. citronellolis* sample. (a) EIC of *m/z* 738.5 that shows the presence of BMP 32:1 and PG 32:1 isomers. (b) MS/MS spectrum of $[M+NH_4]^+$ ion for BMP 32:1. (c) MS/MS spectrum of $[M+NH_4]^+$ ion for PG 32:1. The different characteristic fragment ions at *m/z* 313.27 and 549.48 indicate the structures of BMP and PG, respectively.

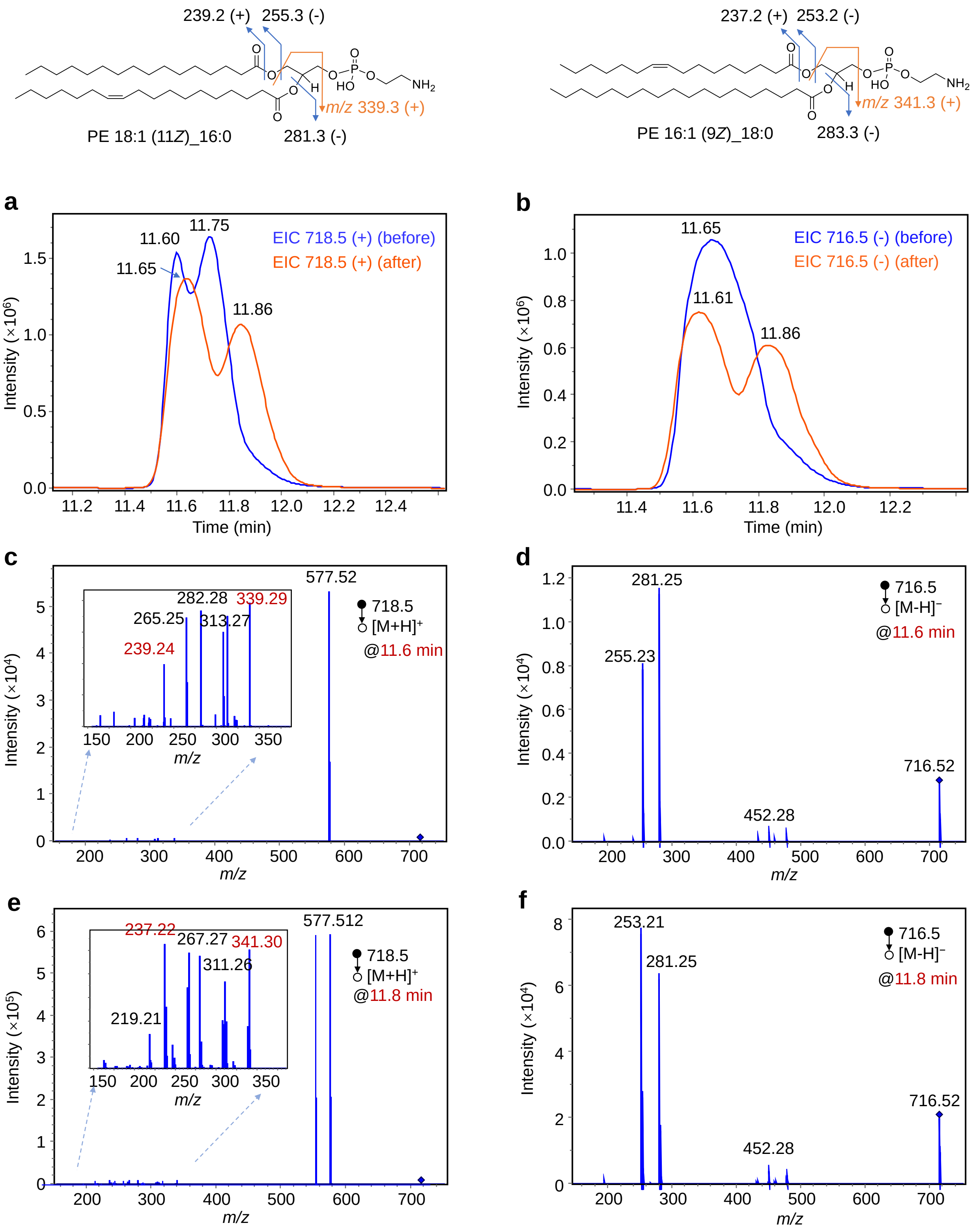

239.2 (+) 255.3 (-)
m/z 339.3 (+)
PE 18:1 (11Z)_16:0
281.3 (-)
237.2 (+) 253.2 (-)
m/z 341.3 (+)
PE 16:1 (9Z)_18:0
283.3 (-)
a
EIC 718.5 (+) (before)
EIC 718.5 (+) (after)
11.60
11.75
11.65
11.86
Intensity (×10^6)
Time (min)
b
EIC 716.5 (-) (before)
EIC 716.5 (-) (after)
11.65
11.61
11.86
Intensity (×10^6)
Time (min)
c
577.52
718.5
[M+H]+
@11.6 min
282.28
339.29
265.25
313.27
239.24
Intensity (×10^4)
m/z
d
281.25
255.23
716.5
[M-H]−
@11.6 min
716.52
452.28
Intensity (×10^4)
m/z
e
577.512
237.22
267.27
341.30
311.26
219.21
718.5
[M+H]+
@11.8 min
Intensity (×10^5)
m/z
f
253.21
281.25
716.5
[M-H]−
@11.8 min
716.52
452.28
Intensity (×10^4)
m/z

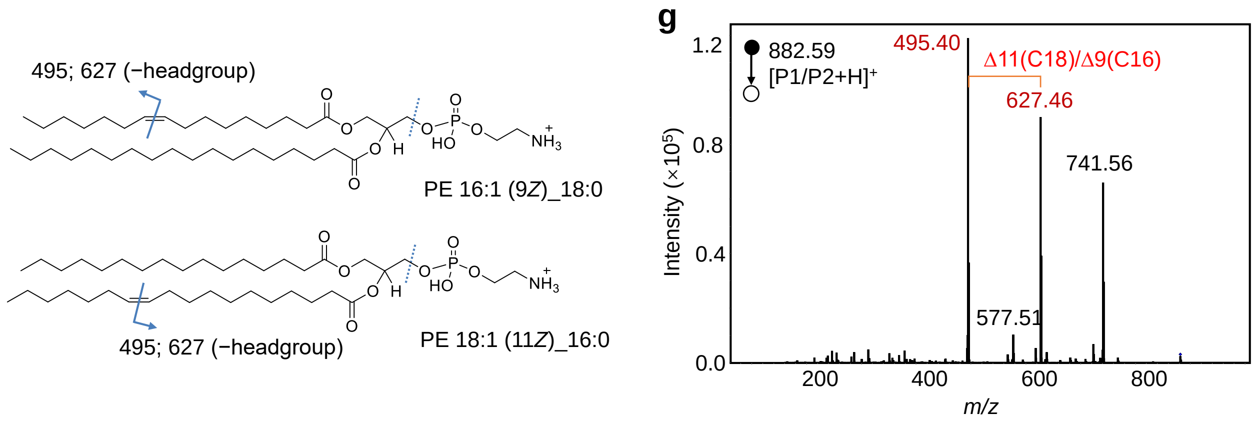

Supplementary Figure 12. Analysis of the structure of PE 34:1 in *P. syringae* bacterial sample. (a) EICs of the protonated ions of PE 34:1 in positive ion mode before and after the photocatalytic reaction. (b) EICs of the deprotonated ions of PE 34:1 in negative ion mode before and after the photocatalytic reaction. The new peak appeared on the right indicated the *cis*-to-*trans* conversion, and confirmed the *Z*-configuration of C=C in these lipids. (c)-(d) MS/MS spectra of (c) $[M+H]^+$ and (d) $[M-H]^-$ for PE 34:1 at retention time of 11.6 min. (e)-(f) MS/MS spectra of (c) $[M+H]^+$ and (d) $[M-H]^-$ for PE 34:1 at retention time of 11.8 min. The neutral loss of 141 Da corresponds to the ethanolamine headgroup indicates the PE subclass of these lipids. The fatty acyl fragments in negative ion modes confirmed the structures of PE 16:1_18:0 and PE 18:1_16:0. (g) Average MS/MS spectrum of the photocycloaddition products. The diagnostic ion pairs at *m/z* 495.40 and 627.46 indicated the isomers of C=C at Δ9 and Δ11, which correspond to the lipid structures of PE 16:1 (9*Z*)_18:0 and PE 18:1 (11*Z*)_16:0. Peak at *m/z* 741.56 corresponds to the fragment of losing ethanolamine headgroup.

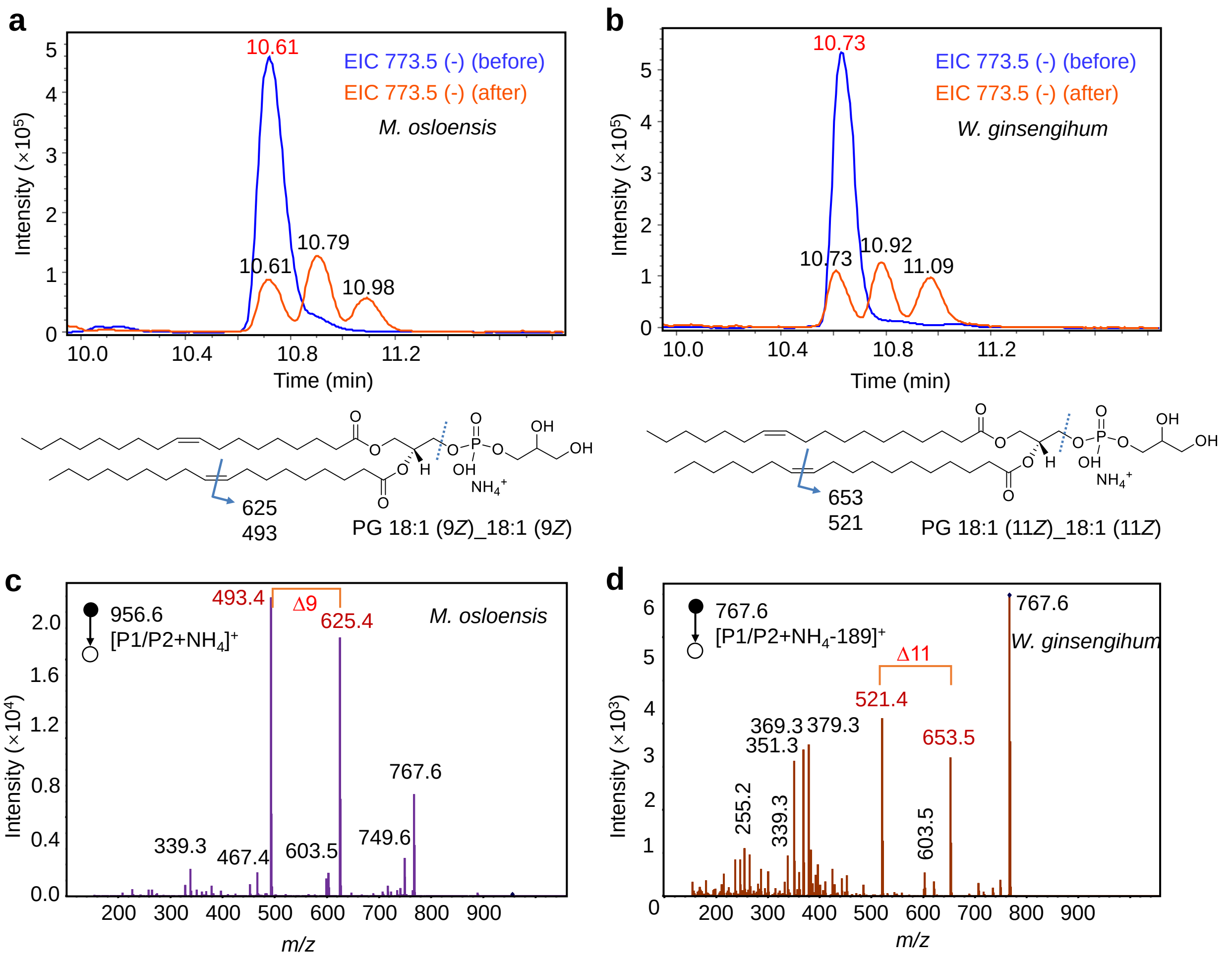

Supplementary Figure 13. Analysis of the structure of PG 36:2 in *M. osloensis* and *W. ginsengihum* bacterial samples. (a)-(b) EICs of the protonated ions of *m/z* 773.5 in negative ion mode before and after the photocatalytic reaction of (a) *M. osloensis* and (b) *W. ginsengihum* samples. The new peak appeared on the right indicated the *cis*-to-*trans* conversion, and confirmed the *Z*-configuration of C=C in these lipids. (c) MS/MS spectrum of the photocycloaddition products of PG 36:2 in *M. osloensis* sample. (d) MS/MS spectrum of ion at *m/z* 767.6, which corresponds to the in-source decay fragment of the photocycloaddition products of PG 36:2 in *W. ginsengihum* sample by losing the head group. The initial formula PG 18:1_18:1 was deduced by the tandem MS spectra in positive and negative ion modes. Based on the diagnostic ion pairs in both samples, only one positional isomer was observed for fatty acyl chains in PG 18:1_18:1. Therefore, the structures were confirmed as PG 18:1 (9*Z*)_18:1 (9*Z*) and PG 18:1 (11*Z*)_18:1 (11*Z*) in *M. osloensis* and *W. ginsengihum*, respectively.

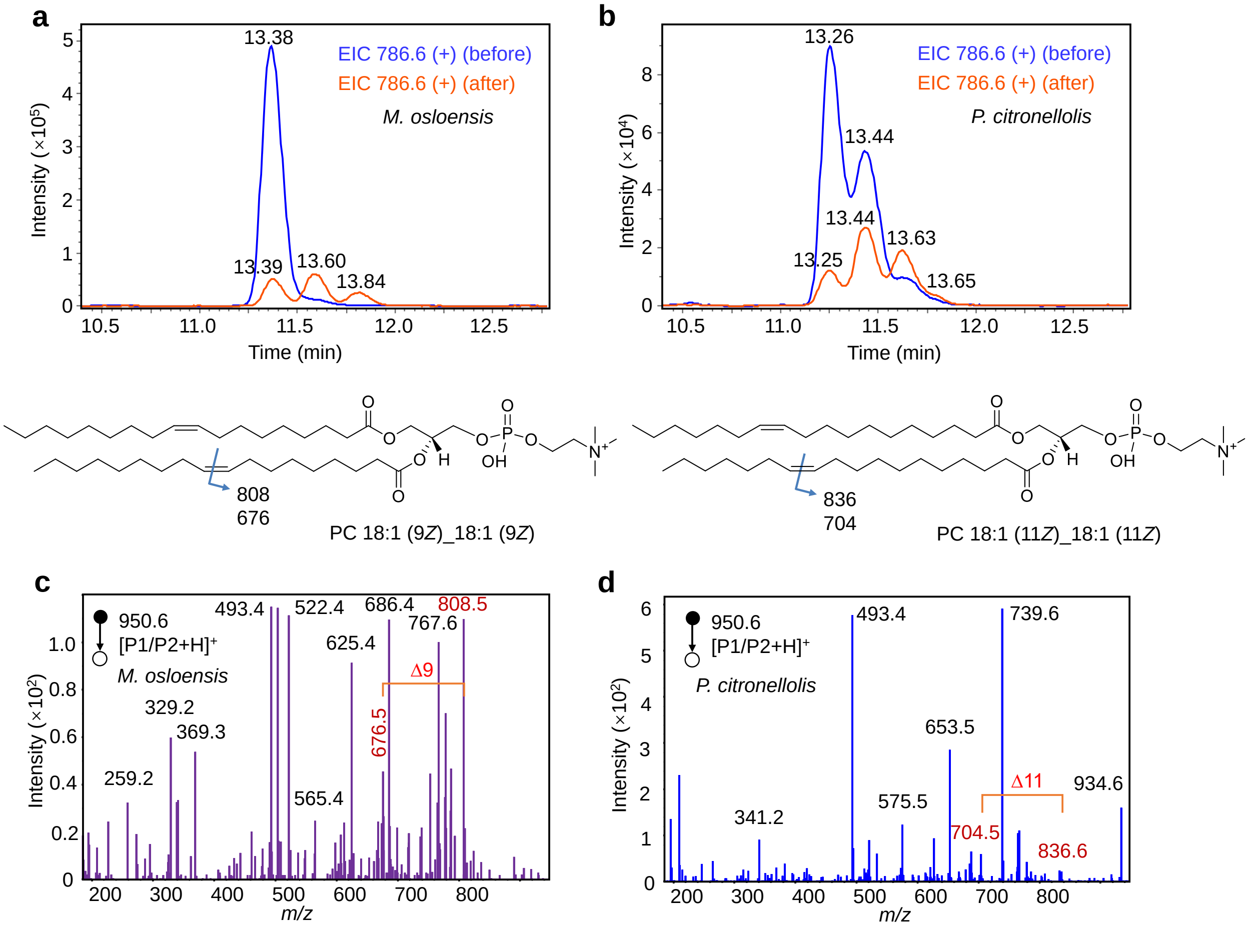

Supplementary Figure 14. Analysis of the structure of PC 36:2 in *M. osloensis* and *P. citronellolis* bacterial samples. (a)-(b) EICs of the protonated ions of *m/z* 786.6 in positive ion mode before and after the photocatalytic reaction of (a) *M. osloensis* and (b) *P. citronellolis* samples. The new peak appeared on the right indicated the *cis*-to-*trans* conversion, and confirmed the *Z*-configuration C=C bond of PC 18:1_18:1 in *M. osloensis*, and both *Z*- and *Z,E*-configuration of C=C bonds of PC 18:1_18:1 in *P. citronellolis* samples, respectively. (c)-(d) MS/MS spectra of the photocycloaddition products of PC 36:2 in (c) *M. osloensis* and (d) *P. citronellolis* samples. The initial formula PC 18:1_18:1 was deduced by the tandem MS spectra in positive and negative ion modes. Based on the diagnostic ion pairs in both samples, the structures were confirmed as PC 18:1 (9*Z*)_18:1 (9*Z*) in *M. osloensis*, and both PC 18:1 (9*Z*)_18:1 (9*Z*) and PC 18:1 (9*Z*)_18:1 (9*E*) in *P. citronellolis*.

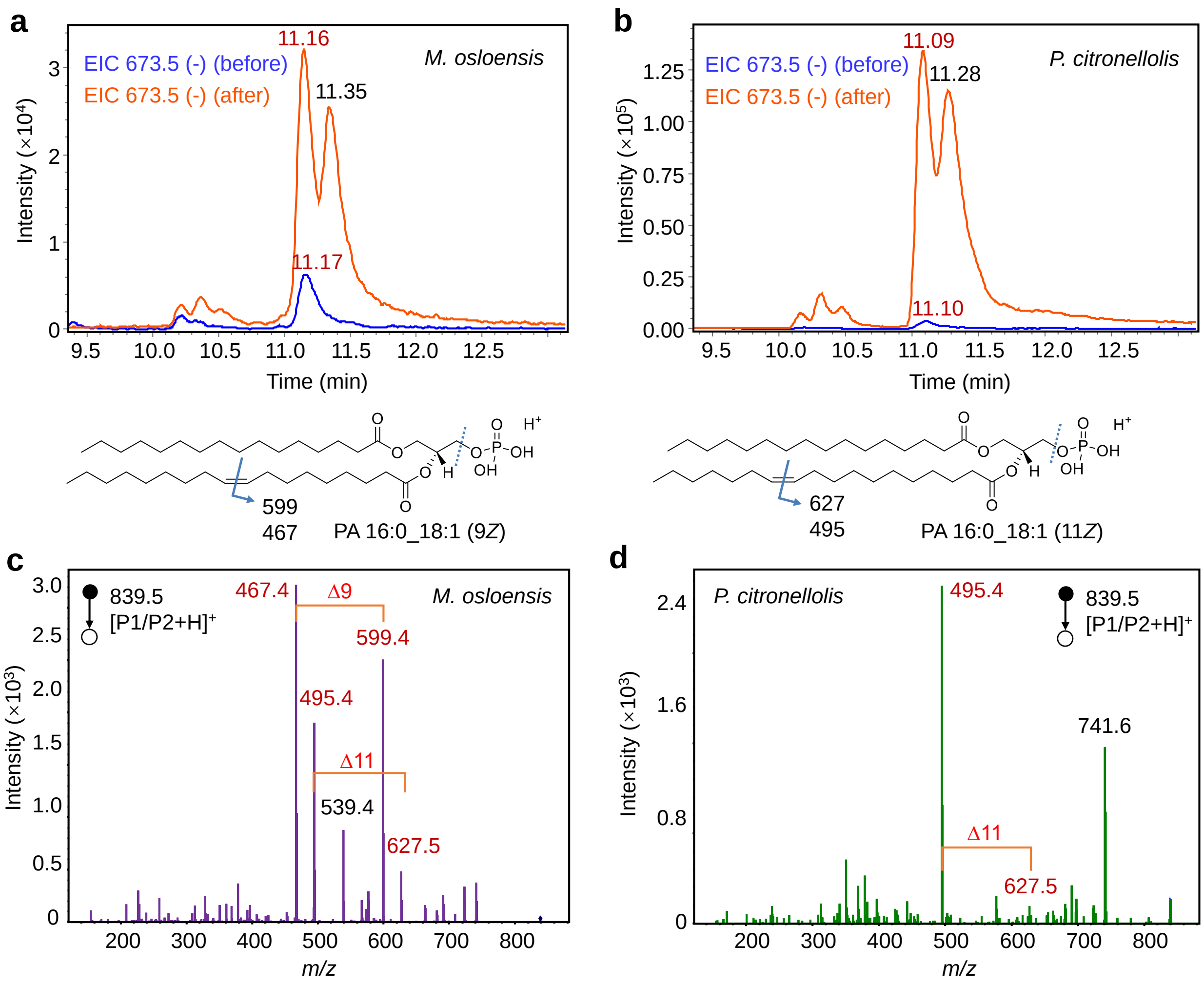

Supplementary Figure 15. Analysis of the structure of PA 34:1 in *M. osloensis* and *P. citronellolis* bacterial samples. (a)-(b) EICs of the protonated ions of *m/z* 673.5 in negative ion mode before and after the photocatalytic reaction of (a) *M. osloensis* and (b) *P. citronellolis* samples. The new peak appeared on the right indicated the *cis*-to-*trans* conversion, and confirmed the *Z*-configuration C=C bonds of PA 16:0_18:1 in both *M. osloensis* and *P. citronellolis* samples. (c)-(d) MS/MS spectra of the photocycloaddition products of PA 34:1 in (c) *M. osloensis* and (d) *P. citronellolis* samples. The initial formula PC 16:0_18:1 was deduced by the tandem MS spectra in positive and negative ion modes. Based on the diagnostic ion pairs in both samples, the structures were confirmed as PA 16:0 _18:1 (9*Z*) and PA 16:0 _18:1 (11*Z*) in *M. osloensis*, and PA 16:0 _18:1 (11*Z*) in *P. citronellolis*.

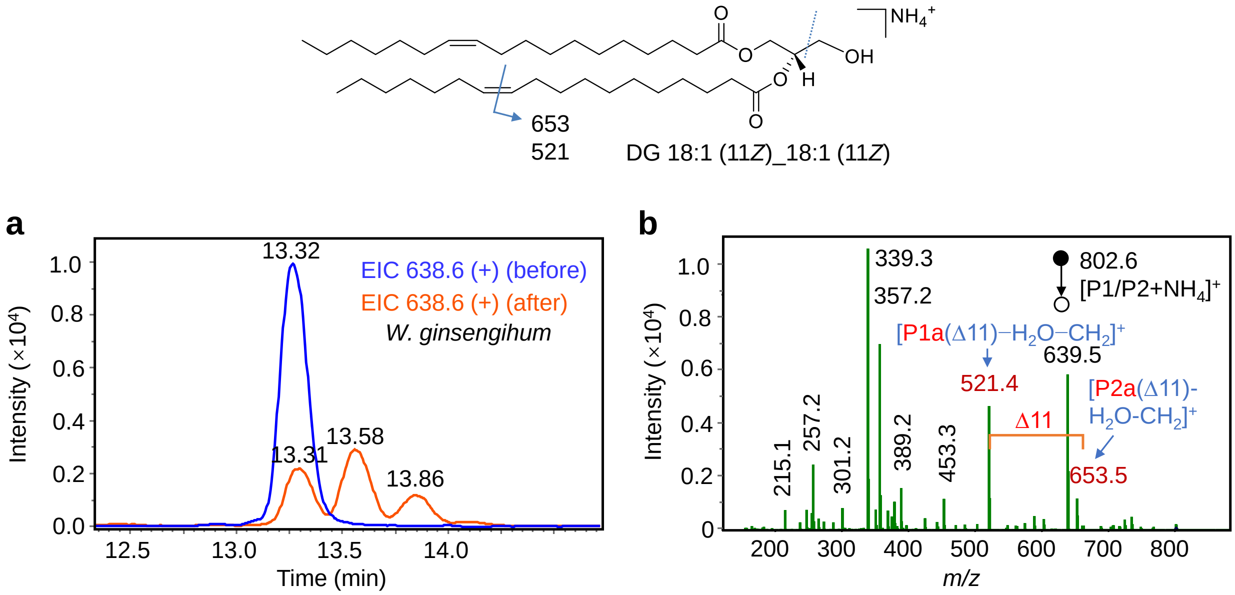

Supplementary Figure 16. Analysis of the structure of DG 36:2 in *W. ginsengihum* bacterial sample. (a) EICs of the ions of *m/z* 638.6 in positive ion mode before and after the photocatalytic reaction. The two new peaks appeared on the right indicated the *cis*-to-*trans* conversion, and confirmed the *Z*-configuration C=C bonds of DG 18:1_18:1. (b) MS/MS spectrum of the photocycloaddition products of DG 36:2 in *W. ginsengihum* sample*.* The initial formula DG 18:1_18:1 was deduced by the tandem MS spectra in positive and negative ion modes. Based on the diagnostic ion pairs in the MS/MS spectrum of the photocycloaddition products, the structures were confirmed as DG 18:1 (11*Z*)_18:1 (11*Z*).

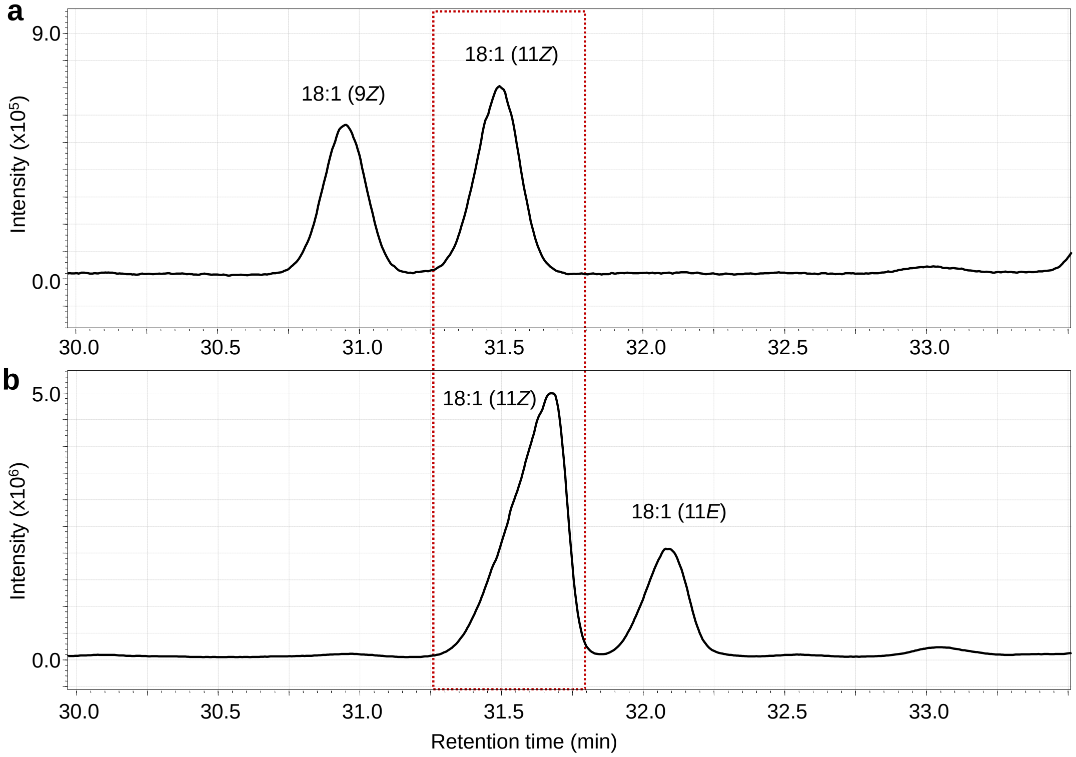

Supplementary Figure 17. GC-MS total ion chromatograms (TIC) of the isomers of FA 18:1. (a) TIC of FA 18:1 (9*Z*) and FA 18:1 (11*Z*). (b) TIC of FA 18:1 (11*Z*) and FA 18:1 (11*E*).

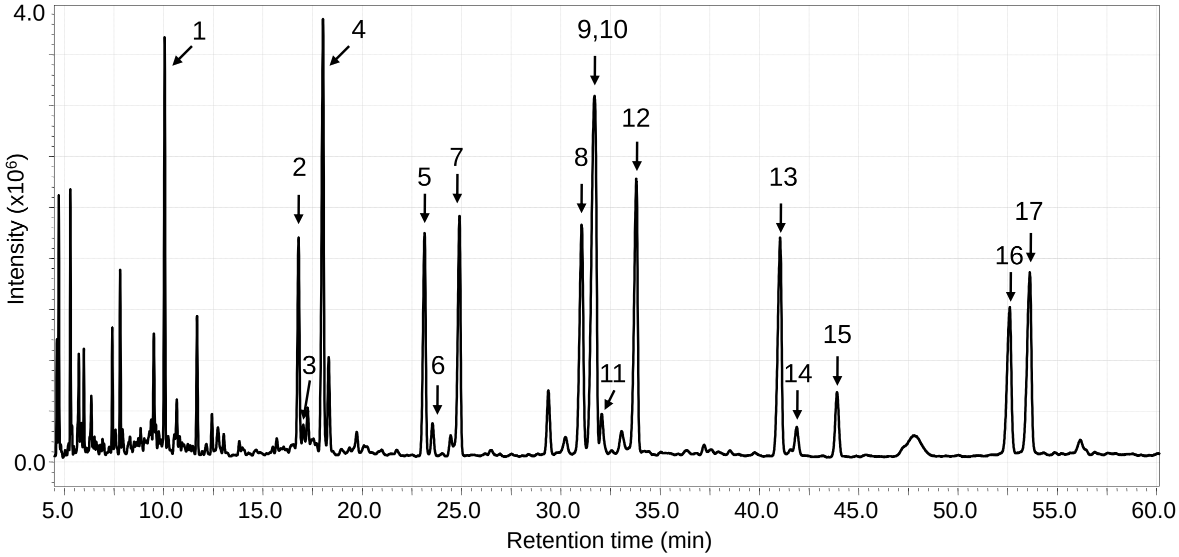

Supplementary Figure 18. GC-MS total ion chromatogram for the analysis of methylated fatty acids standards. **1**: FA 14:0; **2**: FA 16:1 (9*Z*); **3**: FA 16:1 (9*E*); **4**: FA 16:0; **5**: FA 17:1 (10*Z*); **6**: FA 17:1 (10*E*); **7**: FA 17:0; **8**: FA 18:1 (9*Z*); **9**/**10**: FA 18:1 (9*E*)/FA 18:1 (11*Z*); **11**: FA 18:1 (11*E*); **12**: FA 18:0; **13**: FA 19:1 (10*Z*); **14**: FA 19:1 (10*E*); **15**: FA 19:0; **16**: FA 20:1 (11*Z*); **17**: FA 20:1 (11*E*).

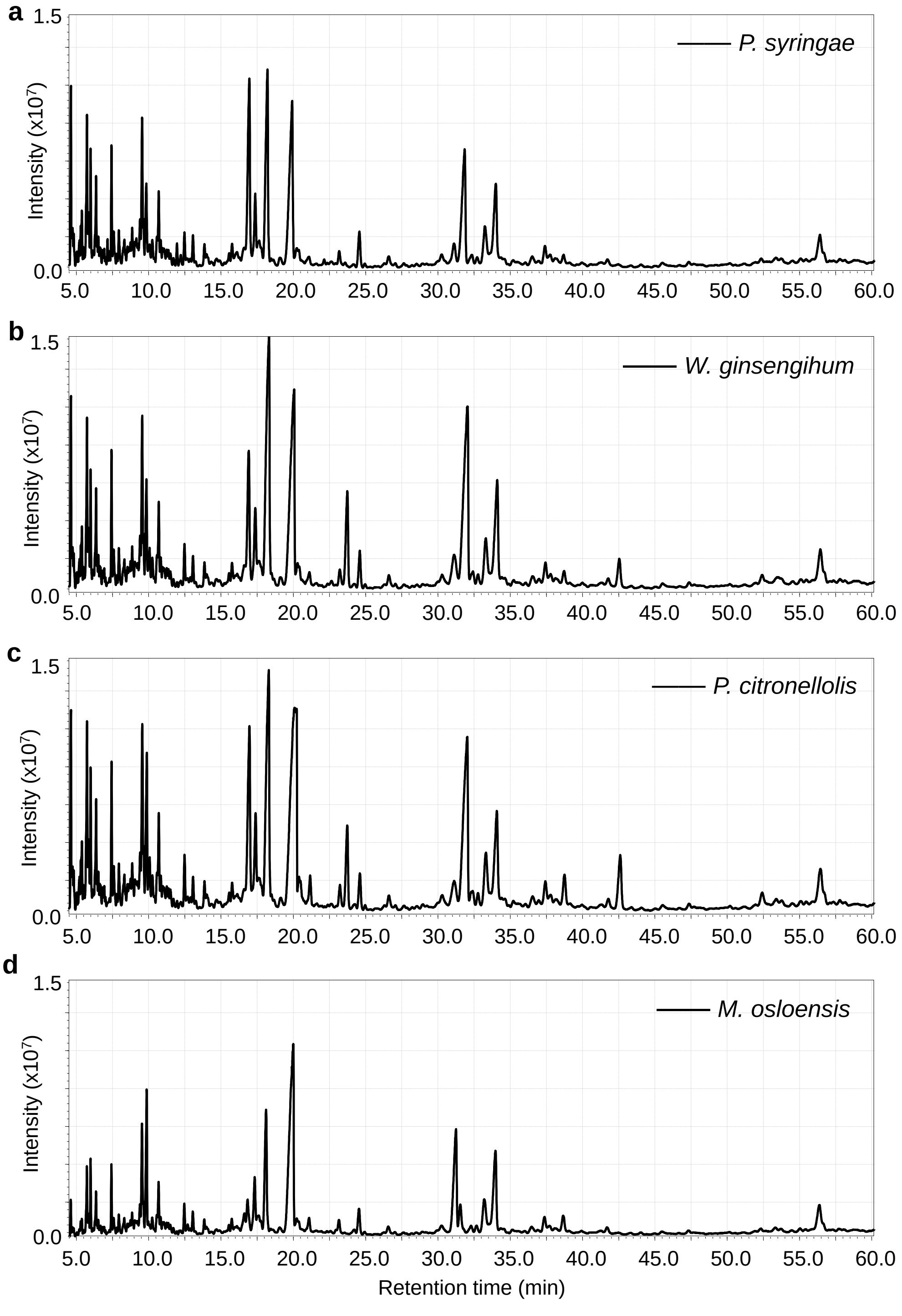

Supplementary Figure 19. GC-MS total ion chromatogram for the analysis of methylated free fatty acids in four bacterial samples. (a) *P. syringae*. (b) *W. ginsengihum*. (c) *P. citronellolis*. (d) *M. osloensis*.

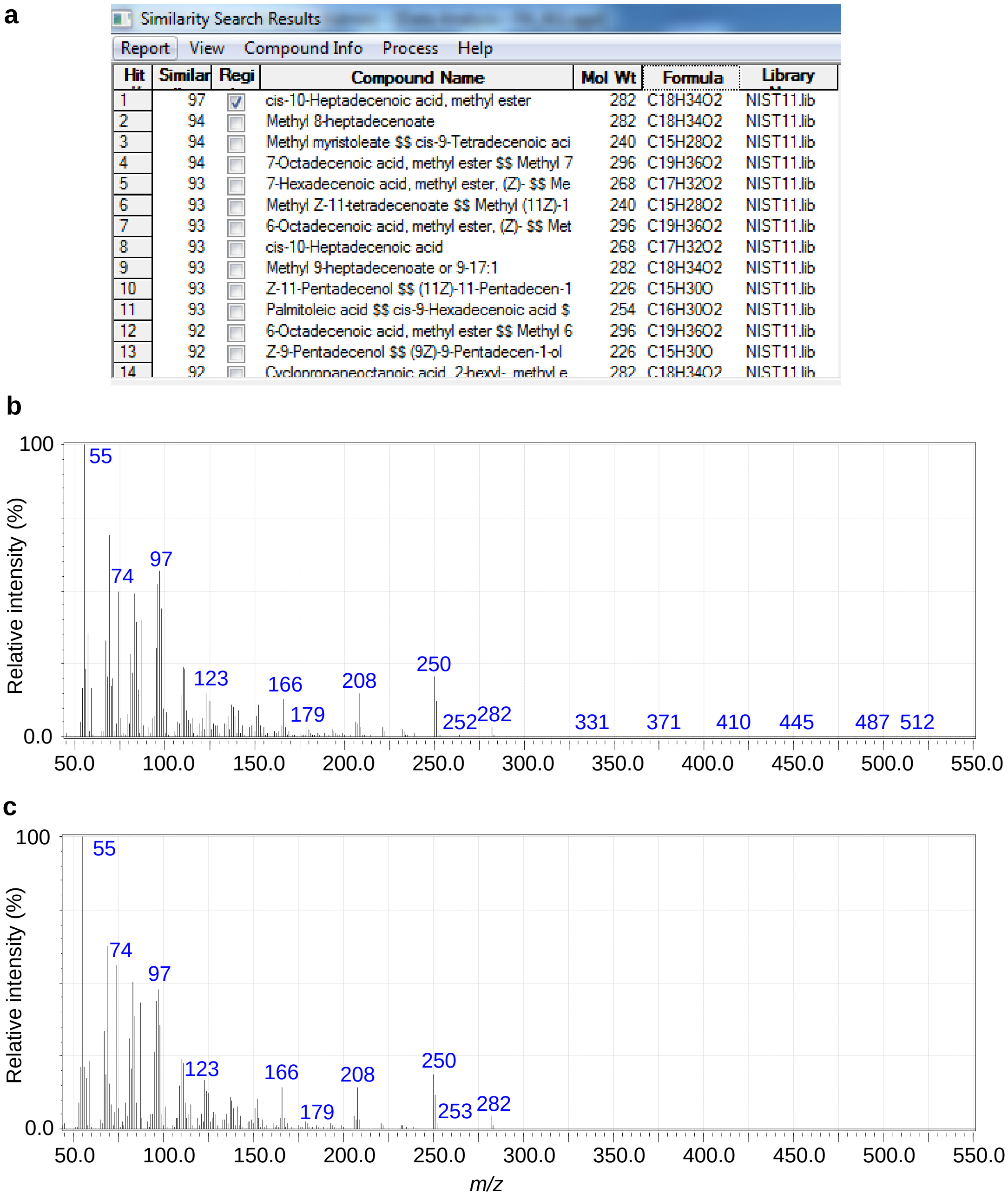

Similarity Search Results

Report View Compound Info Process Help

Hit	Similar	Regi	Compound Name	Mol Wt	Formula	Library
1	97	☑	cis-10-Heptadecenoic acid, methyl ester	282	C18H34O2	NIST11.lib
2	94	☐	Methyl 8-heptadecenoate	282	C18H34O2	NIST11.lib
3	94	☐	Methyl myristoleate $$ cis-9-Tetradecenoic aci	240	C15H28O2	NIST11.lib
4	94	☐	7-Octadecenoic acid, methyl ester $$ Methyl 7	296	C19H36O2	NIST11.lib
5	93	☐	7-Hexadecenoic acid, methyl ester, (Z)- $$ Me	268	C17H32O2	NIST11.lib
6	93	☐	Methyl Z-11-tetradecenoate $$ Methyl (11Z)-1	240	C15H28O2	NIST11.lib
7	93	☐	6-Octadecenoic acid, methyl ester, (Z)- $$ Met	296	C19H36O2	NIST11.lib
8	93	☐	cis-10-Heptadecenoic acid	268	C17H32O2	NIST11.lib
9	93	☐	Methyl 9-heptadecenoate or 9-17:1	282	C18H34O2	NIST11.lib
10	93	☐	Z-11-Pentadecenol $$ (11Z)-11-Pentadecen-1	226	C15H30O	NIST11.lib
11	93	☐	Palmitoleic acid $$ cis-9-Hexadecenoic acid $	254	C16H30O2	NIST11.lib
12	92	☐	6-Octadecenoic acid, methyl ester $$ Methyl 6	296	C19H36O2	NIST11.lib
13	92	☐	Z-9-Pentadecenol $$ (9Z)-9-Pentadecen-1-ol	226	C15H30O	NIST11.lib
14	92	☐	Cyclopropaneoctanoic acid, 2-hexyl-, methyl e	282	C18H34O2	NIST11.lib

Supplementary Figure 20. GC-MS analysis of FA 17:1 (10*Z*) standard. (a) Similarity search results from the NIST library. (b) Measured EI mass spectrum of methylated FA 17:1 (10*Z*) standard. (c) The matched EI mass spectrum of methylated FA 17:1 (10*Z*) in NIST library.

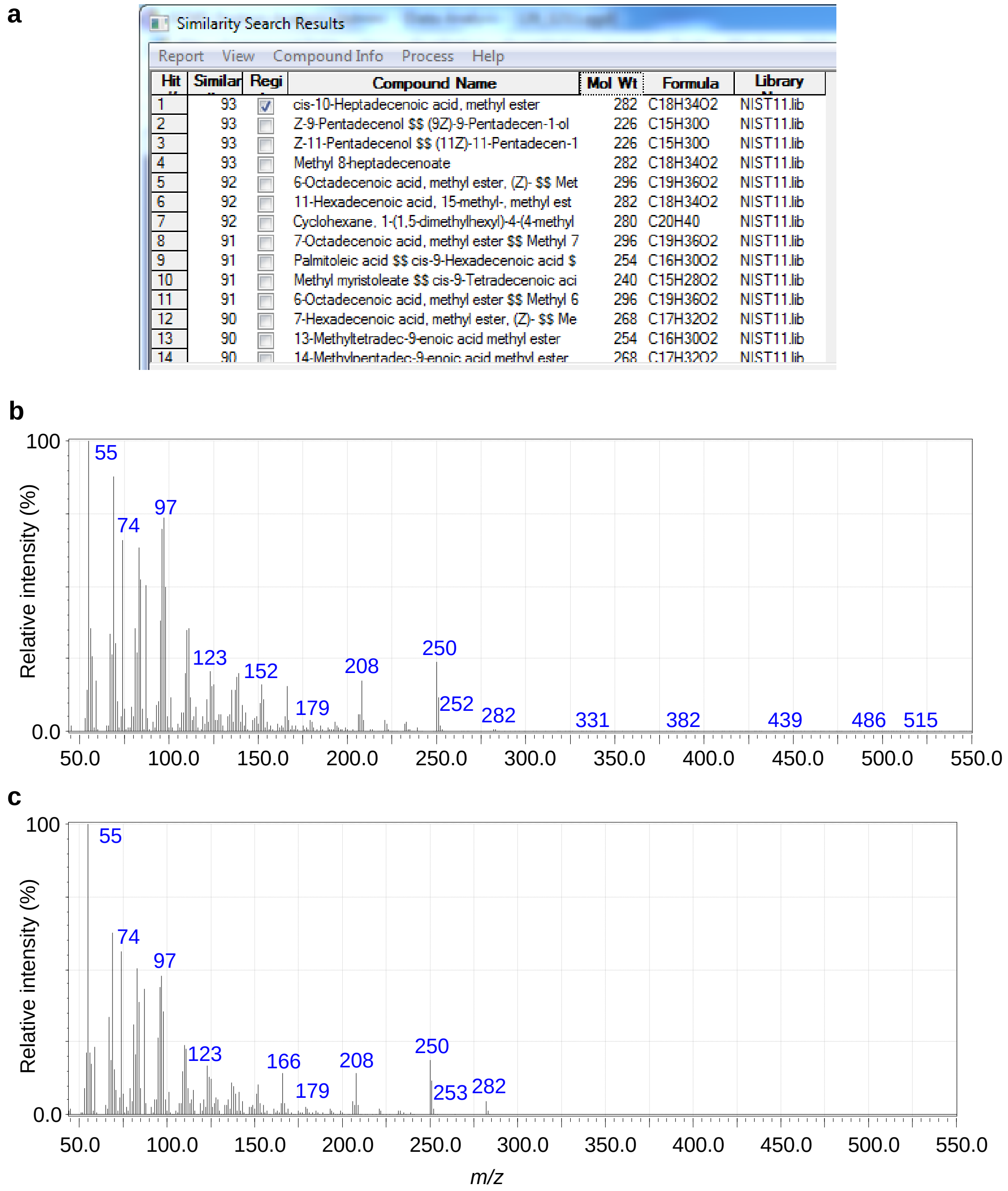
Hit	Similar	Regi	Compound Name	Mol Wt	Formula	Library
1	93	☑	cis-10-Heptadecenoic acid, methyl ester	282	C18H34O2	NIST11.lib
2	93	☐	Z-9-Pentadecenol $$ (9Z)-9-Pentadecen-1-ol	226	C15H30O	NIST11.lib
3	93	☐	Z-11-Pentadecenol $$ (11Z)-11-Pentadecen-1	226	C15H30O	NIST11.lib
4	93	☐	Methyl 8-heptadecenoate	282	C18H34O2	NIST11.lib
5	92	☐	6-Octadecenoic acid, methyl ester, (Z)- $$ Met	296	C19H36O2	NIST11.lib
6	92	☐	11-Hexadecenoic acid, 15-methyl-, methyl est	282	C18H34O2	NIST11.lib
7	92	☐	Cyclohexane, 1-(1,5-dimethylhexyl)-4-(4-methyl	280	C20H40	NIST11.lib
8	91	☐	7-Octadecenoic acid, methyl ester $$ Methyl 7	296	C19H36O2	NIST11.lib
9	91	☐	Palmitoleic acid $$ cis-9-Hexadecenoic acid $	254	C16H30O2	NIST11.lib
10	91	☐	Methyl myristoleate $$ cis-9-Tetradecenoic aci	240	C15H28O2	NIST11.lib
11	91	☐	6-Octadecenoic acid, methyl ester $$ Methyl 6	296	C19H36O2	NIST11.lib
12	90	☐	7-Hexadecenoic acid, methyl ester, (Z)- $$ Me	268	C17H32O2	NIST11.lib
13	90	☐	13-Methyltetradec-9-enoic acid methyl ester	254	C16H30O2	NIST11.lib
14	90	☐	14-Methylpentadec-9-enoic acid methyl ester	268	C17H32O2	NIST11.lib

Supplementary Figure 21. GC-MS analysis of FA 17:1 (10*Z*) in *W. ginsengihum* bacterial sample. (a) Similarity search results from NIST library. (b) Measured EI mass spectrum of methylated FA 17:1 (10*Z*) in *W. ginsengihum* bacterial sample. (c) The matched EI mass spectrum of methylated FA 17:1 (10*Z*) in NIST library.

a

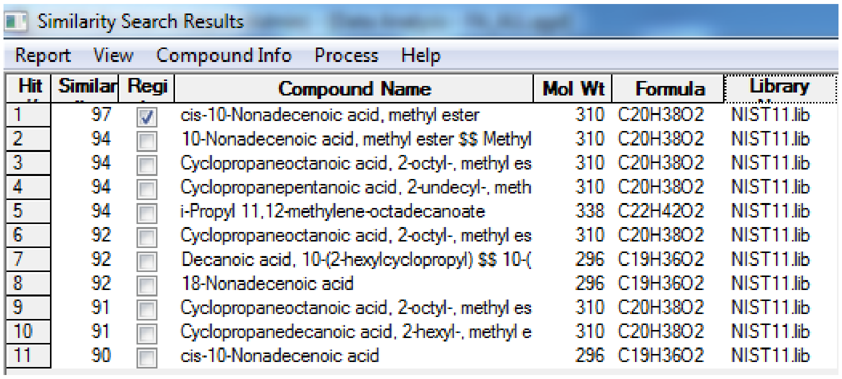

Similarity Search Results

Report View Compound Info Process Help

Hit	Similar	Regi	Compound Name	Mol Wt	Formula	Library
1	97	☑	cis-10-Nonadecenoic acid, methyl ester	310	C20H38O2	NIST11.lib
2	94	☐	10-Nonadecenoic acid, methyl ester $$ Methyl	310	C20H38O2	NIST11.lib
3	94	☐	Cyclopropaneoctanoic acid, 2-octyl-, methyl es	310	C20H38O2	NIST11.lib
4	94	☐	Cyclopropanepentanoic acid, 2-undecyl-, meth	310	C20H38O2	NIST11.lib
5	94	☐	i-Propyl 11,12-methylene-octadecanoate	338	C22H42O2	NIST11.lib
6	92	☐	Cyclopropaneoctanoic acid, 2-octyl-, methyl es	310	C20H38O2	NIST11.lib
7	92	☐	Decanoic acid, 10-(2-hexylcyclopropyl) $$ 10-(	296	C19H36O2	NIST11.lib
8	92	☐	18-Nonadecenoic acid	296	C19H36O2	NIST11.lib
9	91	☐	Cyclopropaneoctanoic acid, 2-octyl-, methyl es	310	C20H38O2	NIST11.lib
10	91	☐	Cyclopropanedecanoic acid, 2-hexyl-, methyl e	310	C20H38O2	NIST11.lib
11	90	☐	cis-10-Nonadecenoic acid	296	C19H36O2	NIST11.lib

b

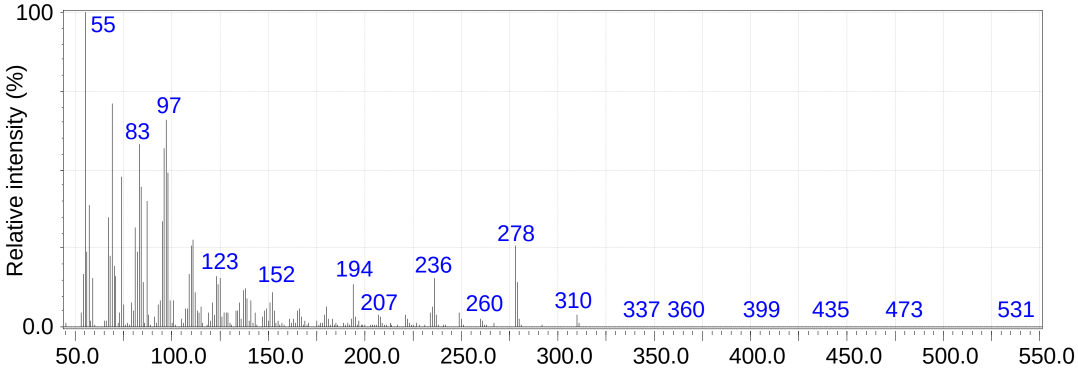

c

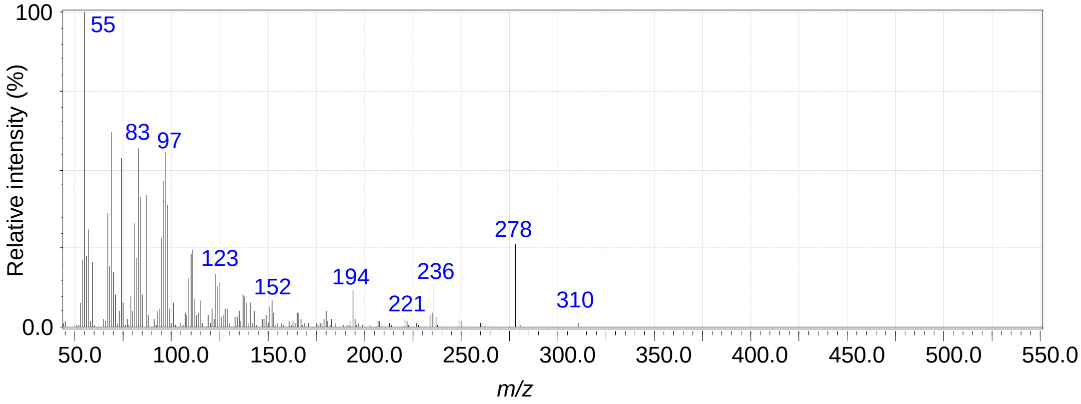

Supplementary Figure 22. GC-MS analysis of FA 19:1 (10*Z*) standard. (a) Similarity search results from the NIST library. (b) Measured EI mass spectrum of methylated FA 19:1 (10*Z*) standard. (c) The matched EI mass spectrum of methylated FA 19:1 (10*Z*) in NIST library.

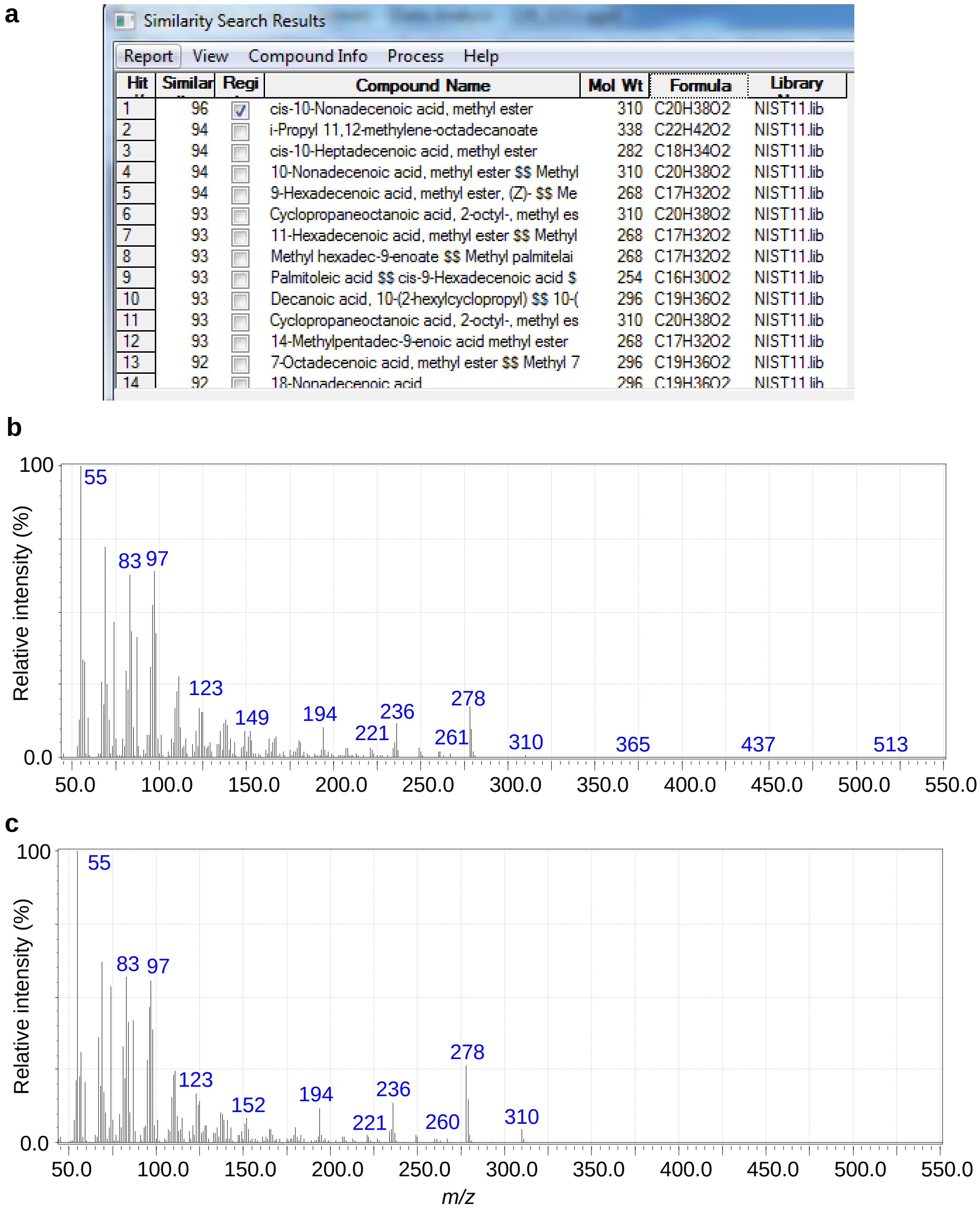

Hit	Similar	Regi	Compound Name	Mol Wt	Formula	Library
1	96	☑	cis-10-Nonadecenoic acid, methyl ester	310	C20H38O2	NIST11.lib
2	94	☐	i-Propyl 11,12-methylene-octadecanoate	338	C22H42O2	NIST11.lib
3	94	☐	cis-10-Heptadecenoic acid, methyl ester	282	C18H34O2	NIST11.lib
4	94	☐	10-Nonadecenoic acid, methyl ester $$ Methyl	310	C20H38O2	NIST11.lib
5	94	☐	9-Hexadecenoic acid, methyl ester, (Z)- $$ Me	268	C17H32O2	NIST11.lib
6	93	☐	Cyclopropaneoctanoic acid, 2-octyl-, methyl es	310	C20H38O2	NIST11.lib
7	93	☐	11-Hexadecenoic acid, methyl ester $$ Methyl	268	C17H32O2	NIST11.lib
8	93	☐	Methyl hexadec-9-enoate $$ Methyl palmitelai	268	C17H32O2	NIST11.lib
9	93	☐	Palmitoleic acid $$ cis-9-Hexadecenoic acid $	254	C16H30O2	NIST11.lib
10	93	☐	Decanoic acid, 10-(2-hexylcyclopropyl) $$ 10-(	296	C19H36O2	NIST11.lib
11	93	☐	Cyclopropaneoctanoic acid, 2-octyl-, methyl es	310	C20H38O2	NIST11.lib
12	93	☐	14-Methylpentadec-9-enoic acid methyl ester	268	C17H32O2	NIST11.lib
13	92	☐	7-Octadecenoic acid, methyl ester $$ Methyl 7	296	C19H36O2	NIST11.lib
14	92	☐	18-Nonadecenoic acid	296	C19H36O2	NIST11.lib

Supplementary Figure 23. GC-MS analysis of FA 19:1 (10*Z*) in *W. ginsengihum* bacterial sample. (a) Similarity search results from NIST library. (b) Measured EI mass spectrum of methylated FA 19:1 (10*Z*) in *W. ginsengihum* bacterial sample. (c) The matched EI mass spectrum of methylated FA 19:1 (10*Z*) in NIST library.

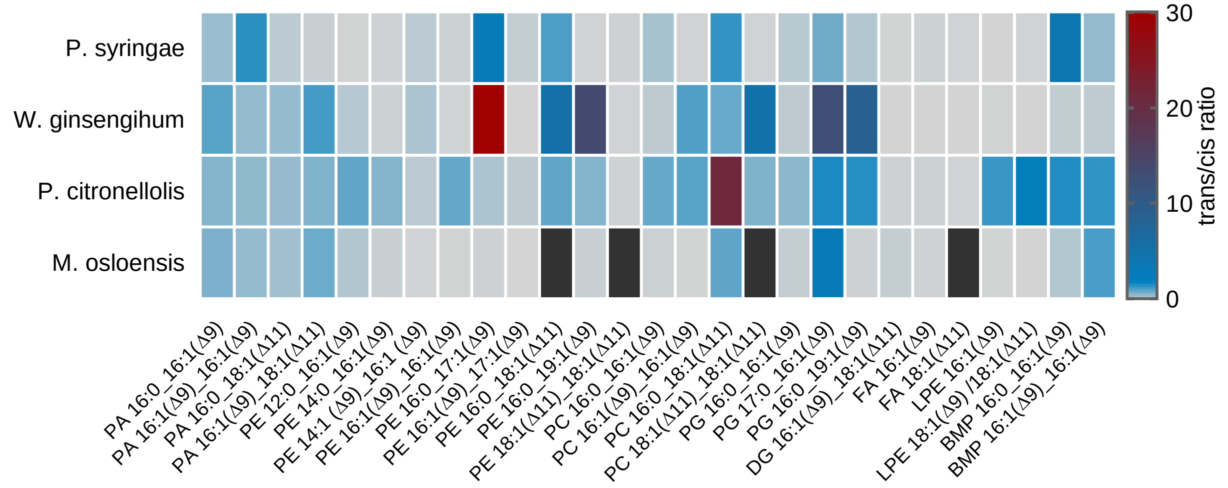

Supplementary Figure 24. Heat map that shows the EIC peak area ratios of lipids *trans*-isomers to their *cis*-counterpart from the four bacterial samples. The black boxes denote the missing data.

Supplementary Table 1. The analytical results of fatty acids in bacteria using GC-MS and LC-MS.

Fatty acids	GC-MS (Peak areas of methylated free FAs in TICs)				LC-MS (Peak areas of FAs in EICs)			
Bacteria	P. syringae	W. ginsengihum	P. citronellolis	M. osloensis	P. syringae	W. ginsengihum	P. citronellolis	M. osloensis
14:0					3.3E+03	8.7E+03	9.7E+04	8.5E+03
16:0	1.5E+08	2.8E+08	2.5E+08	8.2E+07	4.9E+05	8.6E+05	5.7E+05	8.9E+05
16:1 (9*Z*)	1.4E+08	9.8E+07	1.5E+08	2.4E+07	1.6E+05	7.1E+05	1.8E+06	1.0E+06
16:1 (9*E*)					1.1E+04	5.4E+03	1.4E+05	4.2E+04
17:0					3.5E+03	4.7E+03	5.1E+04	1.9E+04
17:1 (10*Z*)	1.2E+07	1.5E+07	1.9E+07	1.1E+07	2.2E+03	7.9E+02	8.5E+04	1.6E+05
17:1 (10*E*)		7.2E+07	6.0E+07		1.2E+04	3.5E+04	2.7E+04	6.1E+03
18:0	9.5E+07	1.3E+08	1.2E+08	8.9E+07	3.1E+05	7.7E+05	1.0E+06	1.0E+06
18:1 (9*Z*)				1.0E+08				2.6E+06
18:1 (9*E*)				2.5E+07				1.4E+04
18:1 (11*Z*)	1.3E+08	2.7E+08	2.5E+08		1.7E+05	8.8E+05	4.0E+06	
18:1 (11*E*)	1.2E+07	1.8E+07	2.3E+07		4.1E+03	5.0E+03	1.4E+05	
19:0					1.2E+03	1.3E+04	9.4E+04	6.5E+02
19:1 (10*Z*)	6.9E+06	1.1E+07	1.4E+07	6.4E+06	3.4E+03	1.5E+02	8.9E+03	7.0E+03
19:1 (10*E*)		2.8E+07	5.7E+07			1.3E+04	6.9E+04	
20:0					5.6E+03	2.3E+04	2.0E+04	1.6E+04
20:1 (11*Z*)					4.0E+03	4.3E+03	4.3E+04	6.4E+03
20:1 (11*E*)							3.9E+03	

Supplementary Table 2. EIC peak area ratios of lipids *trans*-isomers to their *cis*-counterpart from the four bacterial samples

Lipids	P. syringae	W. ginsengihum	P. citronellolis	M. osloensis
PA 16:0_16:1(Δ9)	0.55	1.17	0.76	0.82
PA 16:1(Δ9)_16:1(Δ9)	1.60	0.61	0.63	0.61
PA 16:0_18:1(Δ11)	0.22	0.59	0.58	0.48
PA 16:1(Δ9)_18:1(Δ11)	0.11	1.36	0.77	0.97
PE 12:0_16:1(Δ9)	0.02	0.28	1.09	0.31
PE 14:0_16:1(Δ9)	0.04	0.08	0.76	0.11
PE 14:1 (Δ9)_16:1 (Δ9)	0.23	0.37	0.23	0.03
PE 16:1(Δ9)_16:1(Δ9)	0.08	0.03	1.05	0.02
PE 16:0_17:1(Δ9)	2.98	30.05	0.37	0.06
PE 16:1(Δ9)_17:1(Δ9)	0.15	0.00	0.21	0.00
PE 16:0_18:1(Δ11)	1.27	5.40	1.09	N/A
PE 16:0_19:1(Δ9)	0.03	13.74	0.75	0.12
PE 18:1(Δ11)_18:1(Δ11)[1]	0.06	0.03	0.05	N/A
PC 16:0_16:1(Δ9)	0.43	0.21	1.04	0.08
PC 16:1(Δ9)_16:1(Δ9)	0.03	1.25	1.15	0.01
PC 16:0_18:1(Δ11)	1.51	1.01	21.32	1.10
PC 18:1(Δ11)_18:1(Δ11)[2]	0.03	5.25	0.79	N/A
PG 16:0_16:1(Δ9)	0.26	0.21	0.67	0.15
PG 17:0_16:1(Δ9)	0.95	12.71	1.74	3.17
PG 16:0_19:1(Δ9)	0.30	8.85	1.64	0.06
DG 16:1(Δ9)_18:1(Δ11)	0.02	0.01	0.06	0.16
FA 16:1(Δ9)	0.07	0.01	0.08	0.04
FA 18:1(Δ11)	0.02	0.01	0.03	N/A
LPE 16:1(Δ9)	0.01	0.02	1.46	0.01
LPE 18:1(Δ9) /18:1(Δ11)	0.03	0.01	2.63	0.01
BMP 16:0_16:1(Δ9)	4.09	0.16	1.69	0.31
BMP 16:1(Δ9)_16:1(Δ9)	0.60	0.21	1.53	1.30

Note: [1]ratio of PE 18:1 (11*Z*)_18:1 (11*E*) to PE 18:1 (11*Z*)_18:1 (11*Z*); [2] ratio of PC 18:1 (11*Z*)_18:1 (11*E*) to PC 18:1 (11*Z*)_18:1 (11*Z*). N/A means not detected.

Appendix: Cartesian coordinates and energies of optimized structures

1

M06-2X SCF energy: -856.43105458 a.u.

M06-2X enthalpy: -855.903711 a.u.

M06-2X free energy: -855.982342 a.u.

M06-2X SCF energy in solution: -856.71684890 a.u.

M06-2X enthalpy in solution: -856.189505 a.u.

M06-2X free energy in solution: -856.268136 a.u.

Cartesian coordinates

ATOM	X	Y	Z
C	-0.620363	4.163026	0.453516
C	-1.939955	3.963518	0.463309
H	-0.208787	4.861929	1.182193
C	-2.729939	3.021145	-0.399940
C	-3.300804	1.848906	0.408983
H	-2.119096	2.628225	-1.219610
H	-3.563209	3.565709	-0.865097
C	-4.166669	0.913349	-0.431379
H	-2.469745	1.286579	0.854820
H	-3.892349	2.239451	1.248369
H	-4.994934	1.484391	-0.873872
H	-3.575274	0.528705	-1.274165
C	-4.731026	-0.259973	0.366957
C	-5.599328	-1.195115	-0.472141
H	-5.321137	0.124797	1.210400
H	-3.902529	-0.831251	0.808305
C	-6.163482	-2.369041	0.325120
H	-6.428061	-0.623473	-0.912905
H	-5.009373	-1.579134	-1.316167
H	-5.335173	-2.941926	0.765307
H	-6.752916	-1.985737	1.169946
C	-7.033275	-3.303725	-0.513325
C	-7.589636	-4.472184	0.296365
H	-6.443189	-3.685254	-1.356556
H	-7.860008	-2.729932	-0.951554
H	-6.778610	-5.073929	0.720109
H	-8.204170	-4.112262	1.128384
H	-8.209836	-5.130320	-0.318902
C	0.399450	3.493407	-0.423776

C	1.275005	2.512337	0.366853
H	1.046848	4.256789	-0.876717
H	-0.079239	2.960660	-1.252199
C	2.369302	1.872973	-0.484644
H	1.732404	3.035887	1.217469
H	0.633795	1.731337	0.796333
H	1.910710	1.357209	-1.339996
H	3.007439	2.661202	-0.908189
C	3.234164	0.886245	0.296646
C	4.331918	0.248220	-0.551997
H	2.596345	0.097031	0.717772
H	3.690803	1.400757	1.153310
C	5.188672	-0.739337	0.235974
H	3.875655	-0.264385	-1.410523
H	4.971785	1.037359	-0.971280
H	5.649623	-0.236101	1.093815
H	4.558518	-1.532226	0.655275
C	6.280239	-1.369317	-0.618939
H	6.949943	-0.608923	-1.039451
H	5.858148	-1.902114	-1.480060
C	7.126215	-2.347925	0.156279
O	6.991120	-2.630764	1.319849
O	8.087693	-2.897088	-0.614318
H	8.581143	-3.509473	-0.040849
H	-2.528953	4.512193	1.198866

2

M06-2X SCF energy: -573.08590630 a.u.

M06-2X enthalpy: -572.921763 a.u.

M06-2X free energy: -572.970627 a.u.

M06-2X SCF energy in solution: -573.27062379 a.u.

M06-2X enthalpy in solution: -573.106480 a.u.

M06-2X free energy in solution: -573.155345 a.u.

Cartesian coordinates

ATOM	X	Y	Z
C	2.918128	1.187930	-0.193238
C	1.537056	1.291691	-0.128114
C	0.742146	0.141413	0.051605
C	1.367823	-1.111058	0.183034
C	2.752161	-1.196379	0.106405

C	3.533782	-0.057349	-0.079478
H	3.515548	2.081960	-0.340530
H	1.059359	2.259961	-0.237075
H	0.770515	-1.998351	0.336943
H	3.225289	-2.168595	0.201132
H	4.614447	-0.139490	-0.133933
C	-0.709601	0.319927	0.127956
C	-1.690319	-0.777330	-0.040015
O	-1.114528	1.533792	0.374141
O	-1.334120	-1.926000	-0.174727
C	-3.525348	0.815410	-0.144857
H	-3.099734	1.334681	-1.008395
H	-3.361063	1.399244	0.765215
H	-4.597948	0.687732	-0.296461
O	-3.011525	-0.500625	-0.022749

3

M06-2X SCF energy: -1429.57321516 a.u.

M06-2X enthalpy: -1428.878323 a.u.

M06-2X free energy: -1428.980637 a.u.

M06-2X SCF energy in solution: -1430.03546970 a.u.

M06-2X enthalpy in solution: -1429.340578 a.u.

M06-2X free energy in solution: -1429.442892 a.u.

Cartesian coordinates

ATOM	X	Y	Z
C	0.102157	-0.661432	-0.594688
C	0.604314	0.526130	-1.343699
H	-0.140510	-1.473141	-1.293045
H	0.419525	0.592146	-2.410549
C	1.431594	1.568110	-0.668367
C	2.936366	1.392988	-0.945204
H	1.271773	1.524765	0.416297
H	1.128076	2.570275	-1.001678
C	3.786670	2.472743	-0.280351
H	3.241271	0.402259	-0.586530
H	3.107619	1.404971	-2.030349
H	3.459862	3.462329	-0.629951
H	3.610616	2.455526	0.804089
C	5.280293	2.308020	-0.553213
C	6.134829	3.383006	0.115262

H	5.455831	2.326830	-1.637958
H	5.605298	1.317234	-0.207034
C	7.627959	3.222349	-0.161694
H	5.806252	4.374177	-0.227667
H	5.962060	3.361505	1.200353
H	7.957018	2.230838	0.179723
H	7.801486	3.245149	-1.246833
C	8.483594	4.295845	0.508169
C	9.972984	4.123887	0.220800
H	8.310262	4.270411	1.591709
H	8.152491	5.285270	0.167218
H	10.329103	3.151720	0.577855
H	10.170653	4.173876	-0.855242
H	10.570168	4.899851	0.708245
C	-1.118733	-0.372048	0.284196
C	-2.331592	0.077622	-0.526370
H	-0.841036	0.393053	1.019706
H	-1.358849	-1.280656	0.852015
C	-3.567773	0.303826	0.341717
H	-2.088241	1.004398	-1.063090
H	-2.561143	-0.673722	-1.295464
H	-3.815788	-0.626106	0.871733
H	-3.334721	1.046663	1.117088
C	-4.781466	0.770426	-0.458904
C	-6.018054	0.998719	0.407589
H	-5.014066	0.028106	-1.234798
H	-4.532853	1.700347	-0.988581
C	-7.225878	1.468421	-0.399091
H	-6.268152	0.068195	0.936226
H	-5.784668	1.739586	1.185143
H	-6.985944	2.398049	-0.928065
H	-7.467909	0.733886	-1.175912
C	-8.452847	1.693595	0.474398
H	-8.258722	2.440296	1.254341
H	-8.741373	0.777834	1.005133
C	-9.647178	2.159709	-0.319808
O	-9.673675	2.347318	-1.509879
O	-10.725363	2.353477	0.467174
H	-11.442711	2.650756	-0.119708
O	1.180418	-1.103853	0.273194
C	1.221433	-2.411237	0.600422

C	1.559725	-3.380729	-0.414810
C	1.018780	-2.733569	2.016133
C	2.050078	-2.910669	-1.654396
C	1.422873	-4.774247	-0.231425
O	1.125737	-3.840919	2.510040
O	0.681754	-1.645627	2.738662
C	2.384208	-3.796204	-2.666716
H	2.185212	-1.842003	-1.796841
C	1.760253	-5.648172	-1.254829
H	1.057082	-5.151309	0.714105
C	0.480516	-1.905359	4.122996
C	2.238873	-5.170398	-2.474778
H	2.767560	-3.415316	-3.608182
H	1.647114	-6.716501	-1.099163
H	1.389251	-2.310854	4.574102
H	-0.330406	-2.623584	4.266800
H	0.226274	-0.945127	4.569496
H	2.500410	-5.863128	-3.268297

3a

M06-2X SCF energy: -1429.57193648 a.u.

M06-2X enthalpy: -1428.876686 a.u.

M06-2X free energy: -1428.977493 a.u.

M06-2X SCF energy in solution: -1430.03421095 a.u.

M06-2X enthalpy in solution: -1429.338960 a.u.

M06-2X free energy in solution: -1429.439768 a.u.

Cartesian coordinates

ATOM	X	Y	Z
C	0.858858	-2.111297	-1.328140
C	1.589585	-2.480246	-0.077662
H	0.535775	-2.930915	-1.962171
C	2.310096	-1.323116	0.605442
C	3.560540	-0.901464	-0.167055
H	2.582300	-1.648662	1.614898
H	1.628471	-0.473481	0.729106
C	4.236921	0.329240	0.432763
H	4.273067	-1.737961	-0.182028
H	3.307613	-0.701209	-1.217560
H	3.530452	1.171260	0.423130
H	4.470056	0.136709	1.489143

C	5.512919	0.730090	-0.303897
C	6.189910	1.962282	0.292768
H	5.278416	0.919700	-1.360730
H	6.218106	-0.112622	-0.293129
C	7.467491	2.360511	-0.442671
H	5.485671	2.805944	0.281249
H	6.423090	1.772404	1.349800
H	8.171211	1.516282	-0.432840
H	7.234710	2.552081	-1.499650
C	8.148039	3.590753	0.154591
C	9.423306	3.975957	-0.590764
H	8.379899	3.397365	1.209849
H	7.444115	4.432874	0.144021
H	10.150161	3.157082	-0.568507
H	9.208531	4.199464	-1.641157
H	9.897348	4.857621	-0.150056
C	0.394411	-0.731134	-1.675877
C	-0.842583	-0.261730	-0.885097
H	0.155373	-0.700859	-2.745571
H	1.196378	0.003856	-1.516182
C	-1.409427	1.057777	-1.401023
H	-1.619833	-1.036095	-0.921228
H	-0.567253	-0.145939	0.171500
H	-0.614876	1.817174	-1.424757
H	-1.743494	0.928947	-2.439947
C	-2.572051	1.563905	-0.549645
C	-3.201323	2.844240	-1.094390
H	-2.219581	1.739699	0.476317
H	-3.338111	0.780374	-0.475099
C	-4.340154	3.361919	-0.219635
H	-2.427863	3.619877	-1.187018
H	-3.573584	2.660359	-2.112020
H	-5.111626	2.590686	-0.112155
H	-3.974104	3.564073	0.793509
C	-4.972185	4.627343	-0.784212
H	-5.383829	4.458244	-1.786804
H	-4.231058	5.428369	-0.898862
C	-6.084042	5.155397	0.087059
O	-6.447500	4.680729	1.133214
O	-6.652351	6.258593	-0.442494
H	-7.350303	6.531188	0.178728

O	0.746662	-3.176697	0.910815
C	-0.592384	-3.022549	0.900909
C	-1.385945	-3.936279	0.113714
C	-1.158382	-1.994627	1.782718
C	-0.721895	-4.999746	-0.539067
C	-2.784710	-3.819494	-0.051839
O	-2.340986	-1.743117	1.920406
O	-0.195347	-1.306818	2.432133
C	-1.420631	-5.899039	-1.327607
H	0.347833	-5.110520	-0.396735
C	-3.470747	-4.726005	-0.848331
H	-3.313347	-3.018451	0.446636
C	-0.684208	-0.264485	3.268010
C	-2.800522	-5.766426	-1.490601
H	-0.890564	-6.711793	-1.814423
H	-4.544304	-4.618633	-0.968721
H	-1.340280	-0.667790	4.042645
H	-1.246947	0.466325	2.680333
H	0.197482	0.196019	3.711694
H	-3.347775	-6.470358	-2.109453
H	2.333508	-3.250871	-0.321100

4

M06-2X SCF energy: -1429.57254044 a.u.

M06-2X enthalpy: -1428.877521 a.u.

M06-2X free energy: -1428.979532 a.u.

M06-2X SCF energy in solution: -1430.03593103 a.u.

M06-2X enthalpy in solution: -1429.340912 a.u.

M06-2X free energy in solution: -1429.442923 a.u.

Cartesian coordinates

ATOM	X	Y	Z
C	-2.122196	-0.249729	-0.890359
C	-1.690110	0.788419	-1.865023
H	-2.139685	0.179690	0.121272
H	-1.556286	0.474786	-2.897271
C	-1.374423	2.194343	-1.475998
C	0.126056	2.434925	-1.220125
H	-1.927797	2.462638	-0.565119
H	-1.703929	2.885325	-2.263684
C	0.437376	3.890736	-0.881195

H	0.459420	1.785241	-0.399926
H	0.696539	2.130406	-2.107960
H	0.097822	4.535960	-1.703443
H	-0.142218	4.189664	0.003341
C	1.921786	4.139616	-0.622249
C	2.235080	5.595209	-0.281941
H	2.500116	3.840595	-1.507478
H	2.261513	3.493035	0.198670
C	3.719866	5.846588	-0.028679
H	1.891483	6.242047	-1.101316
H	1.659246	5.892932	0.605489
H	4.064798	5.198847	0.789488
H	4.295893	5.550386	-0.916626
C	4.033917	7.301710	0.313653
C	5.521516	7.538524	0.561270
H	3.459342	7.594992	1.201684
H	3.687111	7.947058	-0.503697
H	5.881547	6.921973	1.391690
H	6.110490	7.277393	-0.324324
H	5.728199	8.584433	0.805431
C	-1.265480	-1.515064	-0.903194
C	0.191510	-1.253708	-0.530710
H	-1.333620	-1.962150	-1.902845
H	-1.710094	-2.238516	-0.207744
C	1.018349	-2.535830	-0.453844
H	0.642827	-0.573249	-1.265694
H	0.237429	-0.734126	0.437711
H	0.573516	-3.211818	0.289272
H	0.963570	-3.060319	-1.417809
C	2.480997	-2.281192	-0.096900
C	3.309852	-3.561510	-0.020382
H	2.534904	-1.755857	0.866594
H	2.925292	-1.604953	-0.840146
C	4.770501	-3.299227	0.337248
H	2.865176	-4.238257	0.722675
H	3.255999	-4.087030	-0.984153
H	5.223012	-2.626074	-0.400111
H	4.834224	-2.778131	1.299520
C	5.587438	-4.582415	0.409320
H	5.567191	-5.125141	-0.543861
H	5.179803	-5.276332	1.154873

C	7.031450	-4.326946	0.761415
O	7.526422	-3.248604	0.972016
O	7.741246	-5.472446	0.819104
H	8.652181	-5.218880	1.049972
O	-3.482559	-0.648714	-1.239514
C	-4.298888	-0.999136	-0.226449
C	-4.749894	0.016727	0.697331
C	-4.755394	-2.391117	-0.199674
C	-4.556340	1.369888	0.342226
C	-5.363963	-0.272695	1.934338
O	-5.569935	-2.848389	0.581048
O	-4.157514	-3.139848	-1.148810
C	-4.947120	2.391225	1.193844
H	-4.123832	1.596691	-0.627634
C	-5.749402	0.759879	2.777888
H	-5.530948	-1.303866	2.215854
C	-4.578521	-4.498622	-1.169704
C	-5.541907	2.091990	2.419972
H	-4.798047	3.424985	0.897296
H	-6.216192	0.522182	3.728677
H	-5.654161	-4.565036	-1.349507
H	-4.353455	-4.987093	-0.218415
H	-4.024482	-4.966819	-1.982036
H	-5.847528	2.890869	3.088060

4a

M06-2X SCF energy: -1429.57486523 a.u.

M06-2X enthalpy: -1428.879843 a.u.

M06-2X free energy: -1428.981637 a.u.

M06-2X SCF energy in solution: -1430.03807846 a.u.

M06-2X enthalpy in solution: -1429.343056 a.u.

M06-2X free energy in solution: -1429.44485 a.u.

Cartesian coordinates

ATOM	X	Y	Z
C	-1.723417	-0.040496	-1.363551
C	-2.387229	0.557055	-0.171586
H	-1.906324	0.422729	-2.328547
C	-2.359694	2.077824	-0.147542
C	-0.941411	2.635246	-0.051361
H	-2.960098	2.421668	0.703713

H	-2.854884	2.445621	-1.055281
C	-0.910869	4.160726	0.018810
H	-0.444886	2.220771	0.837969
H	-0.353423	2.296540	-0.915382
H	-1.407773	4.574898	-0.869350
H	-1.498776	4.497251	0.883898
C	0.504335	4.725669	0.117980
C	0.538028	6.250875	0.188948
H	1.092089	4.388529	-0.747146
H	1.000200	4.309459	1.006019
C	1.952712	6.816101	0.293883
H	0.045022	6.667253	-0.700624
H	-0.052825	6.587668	1.052217
H	2.445754	6.400250	1.183841
H	2.544591	6.479214	-0.568786
C	1.987863	8.341544	0.364678
C	3.407855	8.891654	0.471151
H	1.395538	8.676025	1.226087
H	1.496261	8.755070	-0.525312
H	3.906801	8.511663	1.368940
H	4.008400	8.590541	-0.393741
H	3.414399	9.984264	0.520398
C	-0.842796	-1.241292	-1.260066
C	0.629048	-0.873942	-0.991261
H	-0.897252	-1.834385	-2.181848
H	-1.194479	-1.891134	-0.444933
C	1.531476	-2.099746	-0.873362
H	0.989931	-0.222165	-1.798102
H	0.687134	-0.281618	-0.067437
H	1.160505	-2.746858	-0.066589
H	1.463475	-2.690789	-1.797285
C	2.991859	-1.741568	-0.606742
C	3.895782	-2.966716	-0.486589
H	3.059447	-1.149169	0.316033
H	3.362321	-1.095162	-1.414215
C	5.354031	-2.600638	-0.222475
H	3.526153	-3.612518	0.322173
H	3.826888	-3.560717	-1.408696
H	5.732351	-1.959453	-1.027054
H	5.432315	-2.008276	0.696537
C	6.246204	-3.828939	-0.101895

H	6.212779	-4.440171	-1.012295
H	5.914672	-4.487600	0.710333
C	7.687893	-3.470007	0.157174
O	8.129531	-2.353563	0.260900
O	8.464283	-4.567722	0.265149
H	9.368805	-4.247993	0.429500
O	-3.803383	0.196593	-0.127344
C	-4.050567	-1.131989	-0.068640
C	-3.925188	-1.803239	1.202839
C	-4.461802	-1.787217	-1.315422
C	-3.754544	-1.008904	2.359489
C	-3.952505	-3.208199	1.352073
O	-4.730456	-2.968494	-1.437327
O	-4.516541	-0.922600	-2.348079
C	-3.611215	-1.592159	3.608007
H	-3.765291	0.071232	2.256047
C	-3.805481	-3.777707	2.608859
H	-4.088613	-3.831065	0.478395
C	-4.907711	-1.512996	-3.581596
C	-3.632572	-2.981012	3.740636
H	-3.489482	-0.963186	4.484338
H	-3.825170	-4.858695	2.706697
H	-5.899444	-1.963345	-3.496118
H	-4.197405	-2.288466	-3.879660
H	-4.916750	-0.701312	-4.307732
H	-3.519912	-3.437946	4.718655
H	-1.921032	0.158099	0.743319

5

M06-2X SCF energy: -856.43302143 a.u.

M06-2X enthalpy: -855.905871 a.u.

M06-2X free energy: -855.984259 a.u.

M06-2X SCF energy in solution: -856.71863208 a.u.

M06-2X enthalpy in solution: -856.191482 a.u.

M06-2X free energy in solution: -856.26987 a.u.

Cartesian coordinates

ATOM	X	Y	Z
C	0.510451	-0.109990	0.455765
C	1.489775	0.002077	-0.439726
H	0.608857	0.410870	1.410701

C	2.761738	0.772693	-0.235406
C	4.004430	-0.123817	-0.284572
H	2.724491	1.296313	0.728175
H	2.856493	1.545806	-1.011193
C	5.306055	0.656538	-0.116376
H	3.925518	-0.887369	0.500028
H	4.024393	-0.665215	-1.240515
H	5.377218	1.419635	-0.904148
H	5.282079	1.202238	0.837281
C	6.547633	-0.231722	-0.158829
C	7.850002	0.547758	0.010735
H	6.571966	-0.777227	-1.112546
H	6.475415	-0.994819	0.628599
C	9.091876	-0.339977	-0.028949
H	7.922712	1.310051	-0.777611
H	7.824591	1.094549	0.963740
H	9.020033	-1.102044	0.759840
H	9.117896	-0.887687	-0.981549
C	10.394726	0.439241	0.140087
C	11.626961	-0.460997	0.100804
H	10.366262	0.986647	1.091041
H	10.465427	1.198540	-0.649372
H	11.588938	-1.208816	0.900014
H	11.687458	-0.997689	-0.851952
H	12.550062	0.113083	0.221861
C	-0.761517	-0.880632	0.251086
C	-2.003909	0.016159	0.301231
H	-0.856351	-1.654463	1.026191
H	-0.724436	-1.403307	-0.713041
C	-3.305584	-0.763711	0.131209
H	-2.024225	0.555913	1.258021
H	-1.924654	0.780930	-0.482056
H	-3.282102	-1.306314	-0.824236
H	-3.376579	-1.529104	0.916776
C	-4.546165	0.125590	0.177200
C	-5.848264	-0.653294	0.003770
H	-4.474405	0.891802	-0.606868
H	-4.571541	0.666685	1.133092
C	-7.083637	0.242207	0.050843
H	-5.822866	-1.194503	-0.952491
H	-5.920096	-1.420229	0.787776

H	-7.119324	0.783714	1.003248
H	-7.021500	1.009509	-0.729519
C	-8.375441	-0.544973	-0.124566
H	-8.486727	-1.311839	0.652045
H	-8.389634	-1.085179	-1.079317
C	-9.598434	0.336142	-0.077938
O	-9.602315	1.531041	0.078100
O	-10.733321	-0.376151	-0.234742
H	-11.465675	0.263558	-0.192462
H	1.391212	-0.518912	-1.394622

6

M06-2X SCF energy: -1429.65411120 a.u.

M06-2X enthalpy: -1428.955538 a.u.

M06-2X free energy: -1429.054349 a.u.

M06-2X SCF energy in solution: -1430.11822069 a.u.

M06-2X enthalpy in solution: -1429.419647 a.u.

M06-2X free energy in solution: -1429.518459 a.u.

Cartesian coordinates

ATOM	X	Y	Z
C	-0.521321	-1.380004	-1.265974
C	-2.045220	-1.202638	-1.040738
H	-0.313513	-1.775313	-2.271400
H	-2.647547	-1.223435	-1.954138
C	-2.500680	-0.069389	-0.134539
C	-2.513624	1.291903	-0.829632
H	-1.871220	-0.034745	0.764406
H	-3.513294	-0.296120	0.223492
C	-2.997496	2.413026	0.087976
H	-1.510680	1.538256	-1.200179
H	-3.163515	1.239185	-1.714389
H	-4.002473	2.170027	0.460016
H	-2.347327	2.462812	0.972446
C	-3.026182	3.777755	-0.596671
C	-3.511126	4.899007	0.319884
H	-3.674199	3.726950	-1.482837
H	-2.020063	4.020301	-0.966447
C	-3.535654	6.264955	-0.362358
H	-4.518405	4.657858	0.687265
H	-2.865065	4.947532	1.207560

H	-2.527938	6.507445	-0.728171
H	-4.180182	6.216785	-1.251390
C	-4.022969	7.386444	0.553062
C	-4.039858	8.745762	-0.141423
H	-3.379279	7.432071	1.440927
H	-5.029951	7.143341	0.915848
H	-3.037211	9.019762	-0.486426
H	-4.697751	8.728168	-1.016817
H	-4.392317	9.536105	0.527436
C	0.484015	-0.301603	-0.924761
C	1.902478	-0.869981	-0.860578
H	0.226986	0.156516	0.038442
H	0.435246	0.486846	-1.687058
C	2.943909	0.187049	-0.500660
H	1.926058	-1.682755	-0.125094
H	2.157400	-1.321047	-1.829339
H	2.904388	1.005120	-1.233828
H	2.688877	0.633124	0.470819
C	4.364132	-0.371230	-0.443086
C	5.406482	0.683084	-0.077037
H	4.621055	-0.814030	-1.415101
H	4.403623	-1.191008	0.286980
C	6.824997	0.121140	-0.028559
H	5.362233	1.505679	-0.804738
H	5.151804	1.122627	0.897634
H	6.879643	-0.699811	0.695706
H	7.089755	-0.315167	-0.998610
C	7.853970	1.181726	0.339680
H	7.638820	1.626921	1.318988
H	7.844024	2.015088	-0.373833
C	9.257983	0.633058	0.383626
O	9.578545	-0.506170	0.157022
O	10.155464	1.584322	0.715178
H	11.026006	1.149010	0.719727
O	-0.478758	-2.467420	-0.317341
C	-1.895807	-2.591039	-0.341103
C	-2.486070	-2.794111	1.038475
C	-2.278772	-3.730456	-1.290928
C	-3.868845	-2.829281	1.244990
C	-1.626220	-2.921739	2.129230
O	-1.514586	-4.535566	-1.749878

O	-3.592443	-3.697849	-1.583669
C	-4.379394	-2.992655	2.528690
H	-4.542072	-2.729941	0.399808
C	-2.142154	-3.094499	3.411616
H	-0.556141	-2.884223	1.960041
C	-4.036578	-4.744794	-2.446526
C	-3.518178	-3.129244	3.615520
H	-5.454251	-3.013924	2.679943
H	-1.464322	-3.199748	4.253095
H	-3.841402	-5.718398	-1.992292
H	-3.517252	-4.691152	-3.405661
H	-5.105995	-4.588648	-2.578107
H	-3.919239	-3.260086	4.615705

6a

M06-2X SCF energy: -1429.65450522 a.u.

M06-2X enthalpy: -1428.955927 a.u.

M06-2X free energy: -1429.054029 a.u.

M06-2X SCF energy in solution: -1430.11816431 a.u.

M06-2X enthalpy in solution: -1429.419586 a.u.

M06-2X free energy in solution: -1429.517688 a.u.

Cartesian coordinates

ATOM	X	Y	Z
C	0.774055	1.562997	-0.285845
C	2.141777	1.923620	0.341558
H	0.716107	1.679378	-1.372312
H	2.761475	2.496122	-0.365257
C	2.982121	0.869994	1.026538
C	3.750438	0.007414	0.025439
H	2.346531	0.248225	1.667158
H	3.686295	1.386487	1.691321
C	4.568789	-1.093144	0.697045
H	3.050648	-0.447979	-0.689805
H	4.417942	0.646717	-0.568537
H	5.266048	-0.638778	1.414414
H	3.899079	-1.735571	1.285222
C	5.349565	-1.949703	-0.297233
C	6.173000	-3.047570	0.372857
H	6.015527	-1.305366	-0.887977
H	4.650343	-2.405131	-1.012536

C	6.950217	-3.907016	-0.621762
H	6.874447	-2.591636	1.085439
H	5.507851	-3.690054	0.966595
H	6.248683	-4.365455	-1.333002
H	7.613498	-3.264508	-1.217843
C	7.777764	-5.002828	0.047190
C	8.547349	-5.854426	-0.959247
H	7.114146	-5.642695	0.642940
H	8.478586	-4.543022	0.755805
H	7.862597	-6.344379	-1.659629
H	9.235701	-5.237330	-1.546408
H	9.134131	-6.632960	-0.463446
C	0.153098	0.243454	0.153195
C	-1.244980	0.031832	-0.431398
H	0.104908	0.206518	1.247393
H	0.803996	-0.582993	-0.161863
C	-1.956749	-1.180271	0.164523
H	-1.861070	0.925774	-0.263838
H	-1.169575	-0.080791	-1.522060
H	-1.344783	-2.079364	0.006856
H	-2.034781	-1.047305	1.252752
C	-3.348776	-1.402159	-0.422254
C	-4.069087	-2.606699	0.179621
H	-3.270791	-1.532138	-1.510439
H	-3.957280	-0.500096	-0.265215
C	-5.460401	-2.818109	-0.411689
H	-3.461363	-3.508703	0.022063
H	-4.147283	-2.476584	1.268070
H	-6.074647	-1.923441	-0.256937
H	-5.391039	-2.951130	-1.497551
C	-6.170572	-4.021413	0.194046
H	-6.280792	-3.917414	1.280597
H	-5.598239	-4.944643	0.040652
C	-7.545668	-4.230974	-0.388788
O	-8.065038	-3.547424	-1.234554
O	-8.159962	-5.302342	0.153291
H	-9.031925	-5.363804	-0.274846
O	1.511363	2.826693	1.270747
C	0.312813	2.840419	0.497290
C	0.208575	4.081744	-0.369120
C	-0.889313	2.636120	1.422159

C	-0.640760	4.111801	-1.477817
C	0.986783	5.197882	-0.070276
O	-0.827498	2.092397	2.494219
O	-2.036738	3.071383	0.878665
C	-0.716250	5.249657	-2.272403
H	-1.247884	3.240970	-1.712724
C	0.909794	6.338818	-0.867234
H	1.655190	5.155773	0.783209
C	-3.203891	2.781144	1.645363
C	0.059551	6.367777	-1.967962
H	-1.378300	5.264271	-3.132686
H	1.519708	7.204519	-0.627916
H	-3.132615	3.238069	2.634120
H	-3.317756	1.699357	1.762571
H	-4.038530	3.198129	1.084639
H	0.002745	7.255100	-2.590716

TS-1

M06-2X SCF energy: -1429.54027542 a.u.

M06-2X enthalpy: -1428.846934 a.u.

M06-2X free energy: -1428.947574 a.u.

M06-2X SCF energy in solution: -1430.01024874 a.u.

M06-2X enthalpy in solution: -1429.316907 a.u.

M06-2X free energy in solution: -1429.417548 a.u.

Imaginary frequency: -85.3845 cm-1

Cartesian coordinates

ATOM	X	Y	Z
C	-0.062836	0.477094	2.858408
C	1.298841	0.347031	2.834430
H	-0.464771	1.360280	3.346727
C	2.089639	-0.849284	2.421245
C	3.304600	-0.507863	1.550173
H	1.455537	-1.583794	1.915728
H	2.454421	-1.336375	3.340689
C	4.148056	-1.738129	1.222328
H	2.978072	-0.037184	0.616399
H	3.926180	0.235296	2.067282
H	4.570012	-2.154984	2.147580
H	3.502003	-2.522683	0.801868
C	5.270359	-1.430646	0.233590

C	6.129659	-2.648998	-0.096519
H	5.907986	-0.634957	0.643092
H	4.832160	-1.027434	-0.690014
C	7.247517	-2.342515	-1.090740
H	6.566187	-3.049149	0.829457
H	5.490646	-3.445557	-0.503105
H	6.811152	-1.944032	-2.017161
H	7.886014	-1.544819	-0.685883
C	8.109350	-3.559631	-1.420759
C	9.222056	-3.238531	-2.415361
H	7.470005	-4.355213	-1.824671
H	8.544068	-3.955607	-0.493865
H	8.806762	-2.867486	-3.358295
H	9.887136	-2.463842	-2.019272
H	9.829455	-4.120058	-2.639656
C	-1.072669	-0.568160	2.507308
C	-2.270832	-0.004236	1.738482
H	-1.433999	-1.003111	3.452772
H	-0.615830	-1.382424	1.939059
C	-3.319910	-1.071838	1.440183
H	-2.724589	0.811444	2.317612
H	-1.906454	0.438094	0.806395
H	-2.858834	-1.876645	0.849480
H	-3.658344	-1.534225	2.378443
C	-4.526704	-0.520687	0.683981
C	-5.569635	-1.588501	0.361228
H	-4.187048	-0.049516	-0.248147
H	-4.994566	0.276555	1.277579
C	-6.778011	-1.028203	-0.384563
H	-5.102960	-2.382174	-0.239114
H	-5.902662	-2.065640	1.293785
H	-7.254566	-0.240412	0.210187
H	-6.455579	-0.548698	-1.316011
C	-7.807682	-2.103242	-0.706086
H	-8.170024	-2.596650	0.204401
H	-7.376467	-2.900349	-1.324257
C	-9.006762	-1.553217	-1.436896
O	-9.178492	-0.401033	-1.744861
O	-9.904136	-2.519469	-1.721795
H	-10.638194	-2.081829	-2.187629
O	0.020933	2.078896	1.288579

C	0.688886	2.065134	0.157482
C	0.987711	3.385456	-0.398497
C	1.013031	0.837942	-0.549465
C	0.854436	4.508082	0.443424
C	1.370349	3.592270	-1.738877
O	1.753945	0.741356	-1.516292
O	0.392158	-0.254290	-0.023346
C	1.104627	5.786744	-0.032785
H	0.561388	4.356350	1.475978
C	1.625034	4.876697	-2.199761
H	1.474400	2.744937	-2.402734
C	0.684039	-1.466441	-0.703632
C	1.494083	5.980080	-1.357011
H	1.001048	6.637021	0.634443
H	1.924725	5.017889	-3.233756
H	1.753870	-1.694232	-0.652291
H	0.393960	-1.401808	-1.754841
H	0.105390	-2.240342	-0.195989
H	1.694040	6.979968	-1.729026
H	1.879632	1.201263	3.180621

TS-2

M06-2X SCF energy: -1429.54017180 a.u.

M06-2X enthalpy: -1428.846796 a.u.

M06-2X free energy: -1428.947435 a.u.

M06-2X SCF energy in solution: -1430.01016348 a.u.

M06-2X enthalpy in solution: -1429.316788 a.u.

M06-2X free energy in solution: -1429.417426 a.u.

Imaginary frequency: -84.0797 cm-1

Cartesian coordinates

ATOM	X	Y	Z
C	0.222905	0.483600	2.893121
C	1.582017	0.406911	2.758681
H	-0.179856	1.416444	3.286181
C	2.377397	-0.778501	2.314623
C	3.545707	-0.403941	1.398043
H	1.740984	-1.518331	1.822326
H	2.780919	-1.258225	3.220542
C	4.383426	-1.617975	1.006324
H	3.142169	0.081786	0.504309

H	4.179823	0.338904	1.900811
H	4.760141	-2.117583	1.910301
H	3.741739	-2.350437	0.495430
C	5.559062	-1.260934	0.099284
C	6.390217	-2.474527	-0.311315
H	6.204593	-0.534072	0.611499
H	5.182249	-0.755212	-0.800170
C	7.570454	-2.117916	-1.212346
H	6.761565	-2.983060	0.589560
H	5.744934	-3.198997	-0.827794
H	7.200308	-1.607036	-2.112255
H	8.217870	-1.395673	-0.695442
C	8.399614	-3.331978	-1.626648
C	9.576717	-2.960515	-2.524908
H	7.751623	-4.051169	-2.144033
H	8.767036	-3.841616	-0.726574
H	9.228728	-2.473617	-3.442063
H	10.250885	-2.263636	-2.015740
H	10.158507	-3.841034	-2.812177
C	-0.780211	-0.581394	2.597215
C	-2.022662	-0.062974	1.863186
H	-1.104917	-1.005657	3.561677
H	-0.325434	-1.404168	2.037499
C	-3.068977	-1.154988	1.650460
H	-2.465836	0.762986	2.435335
H	-1.738670	0.355457	0.891512
H	-2.596972	-2.023863	1.168833
H	-3.438128	-1.509270	2.623169
C	-4.240101	-0.681634	0.792628
C	-5.292745	-1.764852	0.568366
H	-3.856824	-0.334964	-0.176969
H	-4.710375	0.190759	1.266595
C	-6.463140	-1.283191	-0.285156
H	-4.821980	-2.634775	0.088863
H	-5.667357	-2.116253	1.540228
H	-6.945663	-0.421546	0.190270
H	-6.098541	-0.926667	-1.255377
C	-7.500373	-2.375380	-0.510234
H	-7.899078	-2.751279	0.440415
H	-7.064081	-3.246385	-1.014492
C	-8.667487	-1.900624	-1.339002

O	-8.818181	-0.787089	-1.773708
O	-9.562800	-2.887391	-1.551372
H	-10.276294	-2.498444	-2.087125
O	1.551986	2.000708	1.174935
C	0.761216	2.084493	0.129271
C	0.597276	3.433507	-0.413673
C	0.179777	0.916982	-0.510948
C	0.999483	4.524624	0.383117
C	0.087376	3.693577	-1.701390
O	-0.675292	0.928260	-1.383753
O	0.690484	-0.253515	-0.037473
C	0.886734	5.825573	-0.084800
H	1.391090	4.332224	1.375437
C	-0.027384	5.001090	-2.153492
H	-0.223840	2.870744	-2.330304
C	0.148836	-1.411080	-0.656755
C	0.370245	6.073413	-1.355434
H	1.197611	6.651543	0.547685
H	-0.428544	5.184288	-3.145666
H	0.323821	-1.393179	-1.734920
H	-0.930052	-1.477758	-0.481624
H	0.661748	-2.260540	-0.202094
H	0.277216	7.091455	-1.720216
H	2.165036	1.221867	3.178216

TS-3

M06-2X SCF energy: -1429.54043241 a.u.
M06-2X enthalpy: -1428.847189 a.u.
M06-2X free energy: -1428.948982 a.u.
M06-2X SCF energy in solution: -1430.01010527 a.u.
M06-2X enthalpy in solution: -1429.316862 a.u.
M06-2X free energy in solution: -1429.418655 a.u.
Imaginary frequency: -113.5348 cm-1

Cartesian coordinates

ATOM	X	Y	Z
C	-0.184645	-1.275907	-0.414799
C	-1.279926	-1.030642	0.362406
H	-0.300173	-1.197855	-1.494271
C	-2.588738	-0.545111	-0.161591
C	-3.745572	-1.494798	0.182758

H	-2.523336	-0.402030	-1.246440
H	-2.797932	0.442414	0.276964
C	-5.091421	-0.975581	-0.318149
H	-3.546568	-2.483569	-0.250275
H	-3.789089	-1.634423	1.271174
H	-5.280161	0.016872	0.113154
H	-5.044081	-0.834153	-1.406639
C	-6.252122	-1.907839	0.022116
C	-7.599191	-1.394604	-0.482470
H	-6.300382	-2.045717	1.111218
H	-6.058535	-2.901741	-0.404971
C	-8.761681	-2.323723	-0.140049
H	-7.791276	-0.399647	-0.057471
H	-7.551197	-1.258413	-1.571877
H	-8.569016	-3.319701	-0.563239
H	-8.811125	-2.458756	0.949591
C	-10.109211	-1.812899	-0.646354
C	-11.261537	-2.751031	-0.296537
H	-10.057999	-1.678922	-1.734532
H	-10.299672	-0.818350	-0.222984
H	-11.103163	-3.742860	-0.733172
H	-11.346589	-2.876523	0.788081
H	-12.217821	-2.370242	-0.666321
C	1.100979	-1.862366	0.070946
C	2.339481	-1.127196	-0.449922
H	1.138899	-2.905185	-0.281287
H	1.108752	-1.893174	1.165131
C	3.636799	-1.786933	0.009592
H	2.308113	-1.095338	-1.547517
H	2.295880	-0.090385	-0.103757
H	3.662232	-1.809182	1.108749
H	3.658524	-2.835818	-0.319186
C	4.881547	-1.069274	-0.507709
C	6.183119	-1.713042	-0.034915
H	4.855816	-0.019633	-0.185608
H	4.861979	-1.053451	-1.606004
C	7.423323	-0.997304	-0.564044
H	6.205243	-1.722557	1.063971
H	6.204091	-2.765707	-0.350638
H	7.413639	-0.989137	-1.660082
H	7.410898	0.054315	-0.255160

C	8.714145	-1.644993	-0.080797
H	8.773984	-2.697056	-0.386268
H	8.775266	-1.649923	1.014523
C	9.942432	-0.944962	-0.605848
O	9.950912	0.015854	-1.332931
O	11.076191	-1.526303	-0.162301
H	11.812248	-1.016838	-0.544471
O	0.016486	0.935247	-0.674581
C	-0.290575	1.788683	0.280267
C	-0.891363	3.038826	-0.170263
C	0.019282	1.539219	1.680936
C	-1.412905	3.091327	-1.480311
C	-0.954786	4.201476	0.626061
O	-0.374477	2.197868	2.629909
O	0.846190	0.470856	1.850079
C	-1.985964	4.255815	-1.969960
H	-1.365880	2.200667	-2.096681
C	-1.538062	5.356375	0.125732
H	-0.555818	4.182658	1.631330
C	1.184941	0.204887	3.203823
C	-2.054952	5.395244	-1.170102
H	-2.386657	4.273631	-2.978941
H	-1.584670	6.241193	0.753243
H	0.293296	-0.038975	3.788429
H	1.671423	1.070396	3.659335
H	1.867769	-0.645447	3.177242
H	-2.506866	6.305452	-1.551434
H	-1.192849	-1.166932	1.440807

TS-4

M06-2X SCF energy: -1429.54052555 a.u.
M06-2X enthalpy: -1428.847291 a.u.
M06-2X free energy: -1428.94914 a.u.
M06-2X SCF energy in solution: -1430.01017712 a.u.
M06-2X enthalpy in solution: -1429.316943 a.u.
M06-2X free energy in solution: -1429.418791 a.u.
Imaginary frequency: -111.8938 cm-1

Cartesian coordinates

ATOM	X	Y	Z
C	0.055299	-0.893428	0.345670

C	-0.992599	-1.231138	-0.461507
H	-0.047297	-1.045558	1.420581
C	-2.232649	-1.931949	-0.010606
C	-3.518179	-1.309631	-0.563510
H	-2.266294	-1.966024	1.083026
H	-2.167640	-2.973186	-0.363526
C	-4.763525	-2.077545	-0.128967
H	-3.572533	-0.271514	-0.222842
H	-3.464541	-1.279778	-1.660360
H	-4.686992	-3.125676	-0.451911
H	-4.811312	-2.097859	0.969488
C	-6.054554	-1.472917	-0.676554
C	-7.306628	-2.223622	-0.228110
H	-6.011634	-1.459798	-1.774483
H	-6.125309	-0.423251	-0.360401
C	-8.597390	-1.623448	-0.780918
H	-7.232127	-3.275031	-0.539700
H	-7.351337	-2.232253	0.870040
H	-8.671024	-0.571191	-0.472296
H	-8.554914	-1.617452	-1.879172
C	-9.850719	-2.370475	-0.328911
C	-11.132443	-1.760434	-0.890514
H	-9.892046	-2.374039	0.767948
H	-9.774769	-3.421328	-0.636870
H	-11.240112	-0.718191	-0.571905
H	-11.122455	-1.772249	-1.985577
H	-12.019282	-2.306843	-0.556942
C	1.329007	-0.289145	-0.140073
C	2.552398	-1.153608	0.199251
H	1.450577	0.698466	0.329805
H	1.270147	-0.120529	-1.221602
C	3.861873	-0.504275	-0.242989
H	2.581408	-1.334211	1.282049
H	2.447023	-2.136481	-0.277689
H	3.825576	-0.311482	-1.323984
H	3.961053	0.476448	0.241693
C	5.085709	-1.358685	0.079434
C	6.398220	-0.707632	-0.351702
H	4.987489	-2.337019	-0.410756
H	5.117423	-1.557751	1.159405
C	7.616401	-1.570471	-0.032834

H	6.364962	-0.502628	-1.430904
H	6.498610	0.268505	0.142884
H	7.656605	-1.781565	1.042059
H	7.527247	-2.544429	-0.527848
C	8.920567	-0.910785	-0.460734
H	9.061397	0.058258	0.033759
H	8.929247	-0.699167	-1.537167
C	10.124819	-1.764235	-0.150957
O	10.104247	-2.849824	0.371837
O	11.271887	-1.166834	-0.533303
H	11.991079	-1.779431	-0.299039
O	-1.379441	0.957609	-0.723822
C	-1.167644	1.829436	0.240165
C	-0.663270	3.127732	-0.191466
C	-1.488741	1.546735	1.632377
C	-0.124993	3.233129	-1.491563
C	-0.708073	4.285338	0.613605
O	-1.170373	2.228449	2.593220
O	-2.231015	0.415056	1.776444
C	0.360336	4.444106	-1.962963
H	-0.089458	2.346700	-2.114792
C	-0.211334	5.487605	0.131653
H	-1.121357	4.226228	1.611487
C	-2.577580	0.111887	3.120468
C	0.323263	5.578856	-1.154241
H	0.775385	4.502445	-2.964598
H	-0.247072	6.368018	0.766049
H	-3.138727	0.933531	3.571224
H	-1.682526	-0.068177	3.722806
H	-3.192316	-0.787902	3.072462
H	0.706855	6.525638	-1.521153
H	-0.856868	-1.136308	-1.537293

FA 18:2 (9*Z*, 12*Z*)

M06-2X SCF energy: -855.20721360 a.u.

M06-2X enthalpy: -854.704428 a.u.

M06-2X free energy: -854.779587 a.u.

M06-2X SCF energy in solution: -855.49405290 a.u.

M06-2X enthalpy in solution: -854.991267 a.u.

M06-2X free energy in solution: -855.066426 a.u.

Cartesian coordinates

ATOM	X	Y	Z
C	-3.904514	-2.178484	-0.610073
C	-5.006049	-1.463087	-0.369464
H	-3.865274	-2.739996	-1.544306
C	-5.299993	-0.591868	0.827589
C	-5.554293	0.848927	0.426688
H	-4.479175	-0.640984	1.550725
H	-6.185829	-0.981736	1.342172
C	-4.620337	1.666622	-0.061683
H	-6.573576	1.216711	0.521916
H	-4.906049	2.679464	-0.346653
C	-3.180500	1.296510	-0.277103
C	-2.194269	2.430127	0.007382
H	-3.045753	0.959596	-1.316643
H	-2.920911	0.433434	0.347544
C	-0.748937	1.984402	-0.202562
H	-2.415441	3.286587	-0.644297
H	-2.326152	2.782182	1.038960
H	-0.538149	1.126180	0.454123
H	-0.630961	1.613877	-1.231945
C	0.283881	3.077632	0.058724
C	1.713073	2.579575	-0.142405
H	0.162246	3.453659	1.082524
H	0.089450	3.927460	-0.607812
H	1.931997	1.747185	0.537436
H	1.858813	2.216860	-1.166277
H	2.449206	3.367164	0.042450
C	-2.670750	-2.291177	0.241052
C	-1.402519	-1.893433	-0.526367
H	-2.558396	-3.326529	0.592812
H	-2.757414	-1.673180	1.141822
C	-0.163788	-1.842082	0.364061
H	-1.238430	-2.598556	-1.352457
H	-1.552756	-0.909094	-0.988502
H	-0.351431	-1.147536	1.196427
H	0.006800	-2.826433	0.822180
C	1.091362	-1.398813	-0.384024
C	2.306650	-1.242371	0.526462
H	0.894685	-0.439151	-0.883741
H	1.319065	-2.119791	-1.181061

C	3.558154	-0.806038	-0.230563
H	2.073914	-0.506828	1.310592
H	2.502109	-2.190056	1.047716
H	3.835057	-1.566364	-0.969807
H	3.351485	0.107529	-0.800588
C	4.737708	-0.554958	0.698867
H	4.977541	-1.444502	1.294718
H	4.512794	0.237266	1.424055
C	5.984759	-0.152925	-0.047654
O	6.087259	-0.041980	-1.243204
O	7.015888	0.078604	0.790888
H	7.772274	0.328613	0.231673
H	-5.794730	-1.477389	-1.120786

TS-5

M06-2X SCF energy: -1428.31345009 a.u.

M06-2X enthalpy: -1427.644598 a.u.

M06-2X free energy: -1427.743933 a.u.

M06-2X SCF energy in solution: -1428.78431657 a.u.

M06-2X enthalpy in solution: -1428.115464 a.u.

M06-2X free energy in solution: -1428.2148 a.u.

Imaginary frequency: -36.8433 cm-1

Cartesian coordinates

ATOM	X	Y	Z
C	1.436897	0.321954	0.966092
C	2.157238	1.368697	0.469624
H	1.553735	0.106637	2.025248
C	2.211656	1.841257	-0.950503
C	1.859149	3.314086	-1.056483
H	1.560022	1.238303	-1.590069
H	3.235352	1.679766	-1.311267
C	0.644676	3.806395	-0.807006
H	2.661044	3.995899	-1.327569
H	0.488832	4.882047	-0.888977
C	-0.550013	2.989462	-0.403553
C	-1.870149	3.478497	-1.001622
H	-0.637612	2.990044	0.694231
H	-0.397588	1.941662	-0.690643
C	-3.038914	2.584747	-0.593220
H	-2.062193	4.510632	-0.678533

H	-1.789339	3.504334	-2.096060
H	-2.839365	1.555477	-0.929190
H	-3.094373	2.540159	0.504910
C	-4.388279	3.036614	-1.145791
C	-5.521396	2.099322	-0.735375
H	-4.330893	3.093104	-2.240186
H	-4.601741	4.053900	-0.794188
H	-5.337068	1.083580	-1.105740
H	-5.605527	2.043481	0.355892
H	-6.486332	2.430576	-1.129435
C	0.421345	-0.502626	0.242613
C	-0.961045	-0.414831	0.906928
H	0.764394	-1.544861	0.249824
H	0.347298	-0.208213	-0.809409
C	-2.039327	-1.130694	0.097326
H	-0.908648	-0.841492	1.917202
H	-1.247679	0.637833	1.030210
H	-2.065163	-0.706482	-0.917255
H	-1.771072	-2.189414	-0.021694
C	-3.426830	-1.017338	0.724349
C	-4.521958	-1.647334	-0.132505
H	-3.666314	0.043770	0.885932
H	-3.421276	-1.486575	1.717584
C	-5.904808	-1.533863	0.503478
H	-4.528279	-1.162232	-1.119726
H	-4.284918	-2.704728	-0.315387
H	-5.923520	-2.067470	1.460631
H	-6.124376	-0.485591	0.738340
C	-7.002649	-2.080788	-0.398655
H	-6.823783	-3.131278	-0.659666
H	-7.042419	-1.539468	-1.352359
C	-8.367395	-1.990771	0.236888
O	-8.607772	-1.549138	1.332028
O	-9.329311	-2.473234	-0.576038
H	-10.169077	-2.379190	-0.092946
H	2.800966	1.902708	1.164815
O	3.196135	-0.837899	0.100445
C	4.466372	-0.700777	0.415798
C	5.403622	-1.267503	-0.553224
C	4.910847	-0.101508	1.666862
C	4.920090	-1.575994	-1.841531

C	6.753340	-1.546386	-0.258354
O	6.066890	0.166727	1.951288
O	3.894537	0.126321	2.544488
C	5.755279	-2.130754	-2.800327
H	3.883176	-1.363037	-2.076486
C	7.579198	-2.095011	-1.229828
H	7.143314	-1.321658	0.724841
C	4.314922	0.690096	3.780714
C	7.091446	-2.392066	-2.502302
H	5.363330	-2.354691	-3.787878
H	8.617870	-2.298522	-0.987530
H	4.803877	1.654704	3.622590
H	5.016497	0.025196	4.289732
H	3.408514	0.813627	4.373467
H	7.746619	-2.822067	-3.253293

TS-6

M06-2X SCF energy: -1428.31188565 a.u.

M06-2X enthalpy: -1427.648621 a.u.

M06-2X free energy: -1427.74611 a.u.

M06-2X SCF energy in solution: -1428.77776435 a.u.

M06-2X enthalpy in solution: -1428.114500 a.u.

M06-2X free energy in solution: -1428.211988 a.u.

Imaginary frequency: -1033.6498 cm-1

Cartesian coordinates

ATOM	X	Y	Z
O	-2.889444	0.199132	-1.627366
C	-2.634802	-0.943462	-1.010833
C	-1.344418	-1.548741	-1.282341
C	-3.673483	-1.585136	-0.200642
C	-0.444781	-0.853236	-2.120311
C	-0.928398	-2.778001	-0.723393
O	-3.499186	-2.485531	0.601667
O	-4.899210	-1.079562	-0.466209
C	0.830603	-1.344291	-2.357734
H	-0.758153	0.084081	-2.567400
C	0.355146	-3.249586	-0.961894
H	-1.606833	-3.335950	-0.091484
C	-5.944700	-1.660348	0.303949
C	1.244363	-2.540847	-1.772676

H	1.509384	-0.782753	-2.993251
H	0.666584	-4.185911	-0.508235
H	-5.730379	-1.568830	1.372034
H	-6.057777	-2.719128	0.059464
H	-6.847306	-1.109460	0.040722
H	2.245809	-2.920958	-1.950051
C	-2.440790	3.662730	-0.327548
C	-3.579511	3.151755	0.140963
H	-2.259314	4.715628	-0.106750
C	-4.186347	1.774078	0.054801
C	-4.269611	1.045337	1.344680
H	-3.594434	1.074355	-0.729572
H	-5.173488	1.825460	-0.417586
C	-3.223887	0.649638	2.095082
H	-5.269036	0.789227	1.692348
H	-3.441020	0.142773	3.035851
C	-1.772539	0.765511	1.744493
C	-0.998724	-0.503904	2.124975
H	-1.339802	1.626256	2.277904
H	-1.650011	0.972385	0.674959
C	0.451243	-0.487250	1.647165
H	-1.024146	-0.620216	3.217834
H	-1.511471	-1.380084	1.707546
H	0.473394	-0.508820	0.547454
H	0.934126	0.453882	1.952899
C	1.256733	-1.668155	2.185511
C	2.679003	-1.707522	1.634160
H	0.735940	-2.599237	1.926545
H	1.282218	-1.619154	3.281852
H	2.659461	-1.852467	0.547921
H	3.203429	-0.765783	1.838684
H	3.263424	-2.521006	2.074803
C	-1.339914	2.983987	-1.086476
C	0.011569	3.125669	-0.370360
H	-1.258184	3.429506	-2.088011
H	-1.573439	1.926408	-1.238730
C	1.077596	2.221957	-0.980291
H	0.336841	4.174691	-0.389802
H	-0.111329	2.860563	0.687916
H	0.707025	1.189467	-0.950574
H	1.215305	2.467442	-2.043648

C	2.421304	2.270473	-0.258537
C	3.366665	1.170851	-0.736301
H	2.254189	2.147619	0.821044
H	2.890210	3.255396	-0.388205
C	4.700103	1.160634	0.005053
H	2.866110	0.200003	-0.605988
H	3.544767	1.280770	-1.816003
H	5.245343	2.093814	-0.177906
H	4.526785	1.121999	1.087366
C	5.570094	-0.021649	-0.399477
H	5.773140	-0.021666	-1.477748
H	5.065746	-0.973950	-0.190139
C	6.896065	-0.036850	0.317872
O	7.270889	0.773347	1.127469
O	7.652252	-1.091499	-0.051747
H	8.480637	-1.024099	0.454762
H	-4.227649	3.828797	0.701322

Supplementary References

[1] Liebisch, G.; Vizcaino, J. A.; Kofeler, H. *J. Lipid Res.* **2013,** *54*, 1523-1530.

[2] Frisch, M. J.; Trucks, G. W.; Schlegel, H. B.; Scuseria, G. E.; Robb, M. A.; Cheeseman, J. R.; Scalmani, G.; Barone, V.; Mennucci, B.; Petersson, G. A.; Nakatsuji, H.; Caricato, M.; Li, X.; Hratchian, H. P.; Izmaylov, A. F.; Bloino, J.; Zheng, G.; Sonnenberg, J. L.; Hada, M.; Ehara, M.; Toyota, K.; Fukuda, R.; Hasegawa, J.; Ishida, M.; Nakajima, T.; Honda, Y.; Kitao, O.; Nakai, H.; Vreven, T.; Montgomery, J. A., Jr.; ; Peralta, J. E.; Ogliaro, F.; Bearpark, M.; Heyd, J. J.; Brothers, E.; Kudin, K. N.; Staroverov, V. N.; Kobayashi, R.; Normand, J.; Raghavachari, K.; Rendell, A.; Burant, J. C.; Iyengar, S. S.; Tomasi, J.; Cossi, M.; Rega, N.; Millam, N. J.; Klene, M.; Knox, J. E.; Cross, J. B.; Bakken, V.; Adamo, C.; Jaramillo, J.; Gomperts, R.; Stratmann, R. E.; Yazyev, O.; Austin, A. J.; Cammi, R.; Pomelli, C.; Ochterski, J. W.; Martin, R. L.; Morokuma, K.; Zakrzewski, V. G.; Voth, G. A.; Salvador, P.; Dannenberg, J. J.; Dapprich, S.; Daniels, A. D.; Farkas, O.; Foresman, J. B.; Ortiz, J. V.; Cioslowski, J.; Fox, D. J. *Gaussian 16*, Revision C.01; Gaussian, Inc.: Wallingford, CT, 2016.

[3] Zhao, Y.; Truhlar, D. G. *Theor. Chem. Acc.* **2007**, *120*, 215-241.

[4] Ribeiro, R. F.; Marenich, A. V.; Cramer, C. J.; Truhlar, D. G. *J. Phys. Chem. B* **2011**, *115*, 14556-14562.

[5] Luchini, G.; Alegre-Requena J. V.; Guan, Y.; Funes-Ardoiz, I.; Paton, R. S. (**2019**). GoodVibes: GoodVibes 3.0.1 http://doi.org/10.5281/zenodo.595246.

[6] Marenich, A. V.; Cramer, C. J.; Truhlar, D. G. *J. Phys. Chem. B* **2009**, *113*, 6378-6396.
