# Supplementary material for: Dual-resolving of positional and geometric isomers of C=C bonds via bifunctional photocycloaddition-photoisomerization reaction system
Source: Nat Commun. 2022 May 12;13:2652. doi: 10.1038/s41467-022-30249-z (PMC9098869; doi:10.1038/s41467-022-30249-z)
Supplement: Supplementary file 3 — Description of Additional Supplementary Information [file 41467_2022_30249_MOESM3_ESM.pdf]

Description of additional Supplementary Information:

(1) Supplementary Information

(2) Supplementary Data 1. Information of the identified lipids in bacterial samples.

(3) Supplementary Data 2. Source data of Figure 5g and Figure 6

(4) Chemical Structures in Figure 1-5
