# Supplementary material for: Dual-resolving of positional and geometric isomers of C=C bonds via bifunctional photocycloaddition-photoisomerization reaction system
Source: Nat Commun. 2022 May 12;13:2652. doi: 10.1038/s41467-022-30249-z (PMC9098869; doi:10.1038/s41467-022-30249-z)
Supplement: Supplementary file 6 — Reporting Summary [file 41467_2022_30249_MOESM6_ESM.pdf]

nature portfolio

Corresponding author(s): Suming Chen

Last updated by author(s): Mar 30, 2022

Reporting Summary

Nature Portfolio wishes to improve the reproducibility of the work that we publish. This form provides structure for consistency and transparency in reporting. For further information on Nature Portfolio policies, see our Editorial Policies and the Editorial Policy Checklist.

Statistics

For all statistical analyses, confirm that the following items are present in the figure legend, table legend, main text, or Methods section.

n/a	Confirmed	
☐	☒	The exact sample size (*n*) for each experimental group/condition, given as a discrete number and unit of measurement
☐	☒	A statement on whether measurements were taken from distinct samples or whether the same sample was measured repeatedly
☐	☒	The statistical test(s) used AND whether they are one- or two-sided<br>*Only common tests should be described solely by name; describe more complex techniques in the Methods section.*
☒	☐	A description of all covariates tested
☒	☐	A description of any assumptions or corrections, such as tests of normality and adjustment for multiple comparisons
☐	☒	A full description of the statistical parameters including central tendency (e.g. means) or other basic estimates (e.g. regression coefficient) AND variation (e.g. standard deviation) or associated estimates of uncertainty (e.g. confidence intervals)
☐	☒	For null hypothesis testing, the test statistic (e.g. *F*, *t*, *r*) with confidence intervals, effect sizes, degrees of freedom and *P* value noted<br>*Give P values as exact values whenever suitable.*
☒	☐	For Bayesian analysis, information on the choice of priors and Markov chain Monte Carlo settings
☒	☐	For hierarchical and complex designs, identification of the appropriate level for tests and full reporting of outcomes
☒	☐	Estimates of effect sizes (e.g. Cohen's *d*, Pearson's *r*), indicating how they were calculated

Our web collection on statistics for biologists contains articles on many of the points above.

Software and code

Policy information about availability of computer code

Data collection	Mass spectra were collected by using Thermo Xcalibur software (version 4.1) in LTQ-Orbitrap Elite mass spectrometer (Thermo Scientific, Germany), and otofControl (version 6.2) in a hybrid trapped ion mobility-quadrupole time-of-flight mass spectrometer (timsTOF Pro, Bruker Daltonics, Bremen, Germany). The acquired GC-MS data were processed with GCMS solution Version 5.2 (Agilent, USA).
Data analysis	(1) Thermo Xcalibur mass spectrometry data system (version 4.1) was used for extract target ion and calculate its peak area. MS-DIAL version 4.12 was used for the process of LC-MS data for qualitative analysis. The raw data was transformed into Abf. files by using IbfConverter 4.60. (2) The GC-MS spectra matching was processed with a standard Library NIST11.(3) All density functional theory (DFT) calculations were carried out using the Gaussian 16 software package. The geometries were optimized using the M06-2X functional with a basis set of 6-31G(d) for all atoms. Vibrational frequency calculations were performed for all the stationary points to confirm if each optimized structure is a local minimum or a transition state structure. Truhlar's quasi-harmonic corrections were applied for entropy calculations with 100 cm−1 as the frequency cutoff using the Goodvibes program (version 3.0.1). Intrinsic reaction coordinate (IRC) calculations were performed to ensure that the saddle points located were transition states connecting the reactants and the products. Solvation energy corrections were calculated in acetonitrile solvent with the SMD continuum solvation model5 based on the gas phase optimized geometries. The M06-2X functional with a basis set of 6-311+G(d,p) for all atoms was used for single-point energy calculations.

For manuscripts utilizing custom algorithms or software that are central to the research but not yet described in published literature, software must be made available to editors and reviewers. We strongly encourage code deposition in a community repository (e.g. GitHub). See the Nature Portfolio guidelines for submitting code & software for further information.

Data

Policy information about availability of data

All manuscripts must include a data availability statement. This statement should provide the following information, where applicable:

- Accession codes, unique identifiers, or web links for publicly available datasets
- A description of any restrictions on data availability
- For clinical datasets or third party data, please ensure that the statement adheres to our policy

The mass spectrometric data generated in this study have been deposited in ProteomeXchange Consortium (https://www.iprox.org/). Project ID: IPX0004110000. Source data are provided with this paper.

Field-specific reporting

Please select the one below that is the best fit for your research. If you are not sure, read the appropriate sections before making your selection.

☒ Life sciences ☐ Behavioural & social sciences ☐ Ecological, evolutionary & environmental sciences

For a reference copy of the document with all sections, see nature.com/documents/nr-reporting-summary-flat.pdf

Life sciences study design

All studies must disclose on these points even when the disclosure is negative.

Sample size	Post hoc power analysis was performed with software G*Power (version 3.1.9.7). In the experiment for comparing the lipid isomer ratios between left and right mouse brain tissues, the results showed that six samples in each group was sufficient to achieve 80% power.
Data exclusions	No data was excluded from analyses.
Replication	In the comparison of the C=C locational isomer ratios between left and right brains of MCAO model mice after reperfusion, six biological replicates were used for each group. All attempts at replication were successful. In the experiments for identifying the lipids in bacterial samples, three replicates for each sample were made and all attempts at replication were successful.
Randomization	The randomization is not applicable to this study. In the experiment of focal ischemia using mice, the lipid isomer ratios were pairwise compared between left and right brain from a single mouse.
Blinding	The preparation of focal ischemia model and the collection of the whole brain tissues from mice were conducted by one investigator, and the dissection of brain tissues and mass spectrometric analysis were performed by another investigator. This investigator knew the classification of the left and right brain tissues, but didn't aware of the exact ischemic side. The bacterial samples were cultured and collected by one investigator, and the lipid extraction and mass spectrometric analysis were performed by another investigator who didn't know the exact names and classifications of these bacterial samples.

Reporting for specific materials, systems and methods

We require information from authors about some types of materials, experimental systems and methods used in many studies. Here, indicate whether each material, system or method listed is relevant to your study. If you are not sure if a list item applies to your research, read the appropriate section before selecting a response.

Materials & experimental systems

n/a	Involved in the study	
☒	☐	Antibodies
☒	☐	Eukaryotic cell lines
☒	☐	Palaeontology and archaeology
☐	☒	Animals and other organisms
☒	☐	Human research participants
☒	☐	Clinical data
☒	☐	Dual use research of concern

Methods

n/a	Involved in the study	
☒	☐	ChIP-seq
☒	☐	Flow cytometry
☒	☐	MRI-based neuroimaging

Animals and other organisms

Policy information about studies involving animals; ARRIVE guidelines recommended for reporting animal research

Laboratory animals	Male C57BL/6 mice (wild-type) (8–10weeks old, 23–25 g) were used in this study.

Wild animals	The study did not involve wild animals.
Field-collected samples	The study did not involve samples collected from fields.
Ethics oversight	All experimental protocols and animal handling procedures were performed in accordance with the National Institute of Health Guide for the Care and Use of Laboratory Animals (NIH Publications No. 80-23) revised 1996 and the experimental protocols were approved by the committee of experimental animals of Tongji Medical College.

Note that full information on the approval of the study protocol must also be provided in the manuscript.
